# Divergent lipid utilization strategies of SARS-CoV-2 and MERS-CoV revealed by comparative multi-omics profiling of infected mouse lung tissues

**DOI:** 10.3389/fimmu.2026.1902981

**Published:** 2026-08-25

**Authors:** Yeonseo Jang, Hyeran Kim, Yufei Li, Jae-Seung Lee, Jaehoon Lee, Han-Woong Lee, Nam-Hyuk Cho, Joo-Youn Cho

**Affiliations:** 1 Department of Clinical Pharmacology and Therapeutics, Seoul National University College of Medicine and Hospital, Seoul, Republic of Korea; 2 Department of Microbiology and Immunology, Seoul National University College of Medicine, Seoul, Republic of Korea; 3 Department of Biomedical Sciences, Seoul National University College of Medicine, Seoul, Republic of Korea; 4 School of Pharmaceutical Sciences, Tsinghua University, Beijing, China; 5 Tsinghua-Peking Joint Center for Life Sciences, Tsinghua University, Beijing, China; 6 Department of Biochemistry, Yonsei University, Seoul, Republic of Korea; 7 Gemcro Inc., Goyang-si, Republic of Korea; 8 Seoul National University, Seoul, Republic of Korea; 9 Institute of Endemic Disease, Seoul National University Medical Research Center, Seoul, Republic of Korea; 10 Seoul National University Bundang Hospital, Seongnam-si, Republic of Korea; 11 Kidney Research Institute, Seoul National University Medical Research Center, Seoul, Republic of Korea

**Keywords:** DIABLO, lipid droplets (LD), lipidomics, MERS-CoV, metabolomics, multi omics, SARS- CoV-2, transcriptomics

## Abstract

**Background:**

Coronaviruses (CoVs), including severe acute respiratory syndrome coronavirus 2 (SARS-CoV-2) and Middle East respiratory syndrome (MERS-CoV), cause respiratory infections with distinct clinical outcomes and case fatality rates. However, the molecular basis of these differences remains unclear. In this study, we sought to define virus-specific host metabolic programs by directly comparing multiomics profiles of the lungs of lethally infected mouse models.

**Methods:**

We performed integrated multiomics analyses, including untargeted metabolomics, transcriptomics, and targeted lipidomics, of lung tissues from human angiotensin-converting enzyme 2 (hiACE2)-human dipeptidyl peptidase 4 (hDPP4) double-knock-in (DKI) mice infected in SARS-CoV-2 or MERS-CoV. Data Integration Analysis and Biomarker discovery using Latent cOmponents (DIABLO) was applied across all three omics layers to identify key distinguishing molecular patterns. Additionally, *in vitro* lipid droplet kinetics were examined in infected Vero E6 cells to validate temporal differences in lipid remodeling.

**Results:**

We identified two distinct strategies for lipid utilization. SARS-CoV-2 infection showed strong activation of energy and amino acid metabolism at an early stage of infection (3 days post infection, DPI), whereas MERS-CoV infection was characterized by sustained alterations in lipid and nucleotide metabolism. Integrative DIABLO analysis of all three omics layers revealed that the key distinguishing features clustered into virus-specific molecular signatures: a triacylglycerol–lipid droplet–interferon axis for SARS-CoV-2 and a phospholipid–sphingolipid–membrane hub for MERS-CoV. *In vitro* lipid droplet kinetics in infected Vero E6 cells confirmed this temporal difference, with SARS-CoV-2 peaking earlier than MERS-CoV.

**Conclusion:**

These findings show that β-CoVs exploit host lipid metabolism through virus-specific and time-dependent remodeling programs, providing a framework for understanding differential pathogenesis and developing host-directed antiviral strategies.

## 1 Introduction

Coronaviruses (CoVs) are enveloped, positive-sense RNA viruses that infect various hosts (1). Among them, severe acute respiratory syndrome coronavirus 2 (SARS-CoV-2) and Middle East respiratory syndrome (MERS-CoV) are two β-CoVs of major public health concern. Both viruses primarily infect the lower respiratory tract and can cause severe pneumonia and acute respiratory distress syndrome (2, 3); however, they enter host cells through different receptors. SARS-CoV-2 binds to angiotensin-converting enzyme 2 (ACE2) (4), whereas MERS-CoV binds to dipeptidyl peptidase 4 (DPP4) (5, 6). This difference in receptor usage leads to distinct tissue tropisms, immune responses, and clinical outcomes.

The two viruses also show markedly different epidemiological patterns. SARS-CoV-2 is highly transmissible but has a relatively low case fatality rate (1–2%), resulting in a pandemic with over 770 million confirmed cases and 7 million deaths (7). In contrast, MERS-CoV spreads inefficiently among humans but is far more lethal, with a case fatality rate of ~34% among over 2,600 confirmed cases since 2012 (7). These differences are usually explained by receptor distribution and immune evasion; however, their metabolic basis remains largely unknown (8). Understanding this dimension is increasingly important, as CoVs continue to evolve through mutations that alter their transmissibility and virulence (9, 10). To better predict the behavior of future variants and emerging coronaviruses, we require mechanistic insights beyond receptor binding and immune escape, particularly into how each virus reprograms the host’s metabolic machinery.

Most molecular studies on coronavirus pathogenesis have focused on clinical observations and immune profiling of patients with COVID-19 (11, 12). Direct comparisons of SARS-CoV-2 and MERS-CoV under controlled experimental conditions are rare. Moreover, ethical and practical constraints make it difficult to study infected human lung tissue directly, leaving host metabolic changes during early pulmonary infection poorly understood at the tissue level. Although recent multiomics approaches have provided valuable insights into host–pathogen interactions (13, 14), most studies have examined a single virus or used blood samples rather than samples from the primary infection site.

In the host systems used by viruses, lipid metabolism is the key interface between viral replication and innate immunity. Viruses reprogram the host lipid pathways to build replication compartments, modify membranes for virion assembly, and secure energy substrates (15, 16). In particular, lipid droplets (LDs), once regarded as simple fat storage organelles, are now known to serve as dynamic signaling platforms that provide fatty acids for viral replication and modulate interferon (IFN)-mediated immune responses (17, 18). Despite this growing recognition, it remains unclear whether different CoVs use host lipid metabolism through distinct temporal strategies, and how such strategies connect to broader metabolic and transcriptional networks.

To address these questions, we performed integrated multi-omics analyses (untargeted metabolomics, transcriptomics, and targeted lipidomics) on lung tissues from human ACE2 (hiACE2)-human DPP4 (hDPP4) double knock-in (DKI) mice infected with SARS-CoV-2 or MERS-CoV (19). Using Data Integration Analysis and Biomarker discovery using Latent cOmponents (DIABLO)-based multiomics integration and correlation network analysis, we aimed to define virus-specific molecular signatures at the tissue level, characterize the temporal patterns of lipid remodeling during early infection, and identify key molecular axes linking metabolic reprogramming to distinct pathological outcomes.

Our findings revealed that SARS-CoV-2 and MERS-CoV employed fundamentally different, temporally programmed lipid utilization strategies. SARS-CoV-2 triggered rapid triacylglycerol (TG) accumulation and LD biogenesis, which are linked to coordinated changes in energy metabolism and innate immune gene expression. In contrast, MERS-CoV drove a delayed but progressive phospholipid and sphingolipid membrane remodeling program. These findings provide new insights into virus-specific metabolic reprogramming and suggest potential targets for host-directed antiviral therapies.

## 2 Methods

### 2.1 Chemicals and reagents

Water, methanol (MeOH, 99.8%), acetronitrile (ACN, 99.8%), and isopropanol in high-performance liquid chromatography grade were purchased from J.T. Baker (Phillipsburg, NJ, USA). Formic acid (95%), ethanol (EtOH), methyl tert-butyl ether (MTBE), and Ammonium Acetate (AmAc) were purchased from Sigma-Aldrich (St. Louis, MO, USA). 4,4Difluoro-1,3,5,7,8-Pentamethyl-4-Bora-3a,4a-Diaza-s-Indacene (BODIPY™493/503) and TRIzol™ were purchased from Invitrogen (Waltham, MA, USA).

### 2.2 Animal model and infection procedures

hiACE2 transgenic mice were crossed with hDPP4 knock-in mice to generate hiACE2–hDPP4 DKI mice (19, 20). Infection experiments were performed using these hiACE2–hDPP4 DKI mice (**Supplementary Figure 1A**). All mice were anesthetized by intraperitoneal injection of ketamine (90 mg/kg; Yuhan Co., Korea) and xylazine (15 mg/kg; Elanco, USA). Mice were then intranasally inoculated with either 1 × 10^5^ focus-forming units (FFU) of SARS-CoV-2 (GenBank accession no. OR145098.1) or 1 × 10^4^ FFU of MERS-CoV (GenBank accession no. MT576585.1). At 3 and 6 days post-infection (dpi), mice were euthanized by carbon dioxide (CO_2_) inhalation in accordance with the AVMA Guidelines for the Euthanasia of Animals. Animals were placed in a 6-L euthanasia chamber, and 100% CO_2_ was delivered at a flow rate of 3 L/min (50% chamber volume displacement per minute) using a calibrated flow meter. Gas flow was maintained until respiratory arrest was confirmed. Plasma and lung tissues were subsequently collected for downstream analyses. Viral RNA quantification in the lung tissues confirmed successful infection in both groups (**Supplementary Figure 1B**). All animal experiments were approved by the Institutional Animal Care and Use Committee (IACUC) of Seoul National University Bundang Hospital (approval number: BA-2308-374-007-11) and were conducted in accordance with the relevant ethical guidelines. All experiments involving infectious viruses were conducted in the certified Biosafety Level 3 (BSL-3) facility at Seoul National University College of Medicine.

### 2.3 Quantification of viral loads

Quantification of viral RNA in mouse lungs was performed by first extracting RNA using TRIzol (Invitrogen), according to the manufacturer’s guidelines. cDNA synthesis was conducted with 1 μg of the extracted RNA employing reverse transcriptase PCR (iNtRON Biotechnology, Seongnam, South Korea), following the provided instructions. The ORF1 regions of SARS-CoV-2 and MERS-CoV were then targeted for qRT-PCR analysis using the CFX96TM Real-Time PCR Detection System (Bio-Rad, Hercules, CA, USA). To determine viral RNA copy numbers, standard DNA fragments within the ORF1 region of SARS-CoV-2 and MERS-CoV were synthesized using specific primer pairs: 5’-CCTCACTTGTTCTTGCTCGC-3’ (forward) and 5’-ACACACAACAGCATCGTCAG-3’ (reverse) for SARS-CoV-2; 5’-CGAGTGATGAGCTTTGCGTGA-3’ (forward) and 5’-CCTTATGCATAAGAGGCACGAG-3’ (reverse) for MERS-CoV. Sample viral loads were quantified by comparing qRT-PCR results to standard curves derived from serial dilutions of the standard DNA. Data analysis was conducted using Bio-Rad CFX Manager Software (version 3.1; Bio-Rad).

### 2.4 *In vitro* lipid droplet immunofluorescence analysis

Vero E6 cells were seeded into 24-well plates at 80% confluence and incubated overnight. Cells were infected with SARS-CoV-2 (BetaCoV/Korea/KCDC03/2020) or MERS-CoV (CoV/KOR/KNIH/002_05_2015; Permission No. 1-001-MER-IS-2015001) at a multiplicity of infection (MOI) of 0.1 for 1 h at 37 °C. Following infection, the inoculum was removed, and the cells were washed with phosphate-buffered saline (PBS) before adding maintenance media (DMEM supplemented with 2% FBS). Cells were fixed with 4% paraformaldehyde at 1, 6, 12, 18, and 24 h post-infection (HPI).

Fixed cells were washed three times with PBS and permeabilized with 0.1% Triton X-100 in PBS for 10 min. Subsequently, the cells were incubated overnight at 4 °C with primary antibodies against SARS-CoV-2 or MERS-CoV nucleocapsid protein (NP), followed by incubation with Alexa Fluor 633–conjugated anti-rabbit IgG for 1 h. Lipid droplets (LDs) were stained with 1 ng/mL BODIPY 493/503 for 30 min, and nuclei were counterstained with 4′,6-diamidino-2-phenylindole (DAPI) for 5 min. Coverslips were mounted onto glass slides using an antifade mounting medium. Fluorescence images were acquired using an Olympus FV3000 confocal microscope (Tokyo, Japan), and the number and diameter of LDs were quantified using ImageJ software.

For viral inactivation, 1 mL of the viral suspension was placed in a 60-mm dish and exposed to ultraviolet (UV) light for 1 h. To ensure uniform UV exposure, the dish was gently rocked every 15 min. The UV-inactivated virus was subsequently stored at −80°C until further use.

### 2.5 Lung tissue sample preparation

Lung tissue samples were prepared at the certified Biosafety Level 3 (BSL-3) facility at Seoul National University College of Medicine. Lung tissues from infected mice were harvested and immediately snap-frozen in liquid nitrogen within 30 s to preserve tissue integrity. The frozen lung tissue samples from 25 mice that had been subjected to gamma irradiation for viral inactivation were thawed on ice and cut into small pieces. Approximately 100 mg of tissue was weighed for each sample and transferred into 1.2-mL tubes. Ice-cold 80% MeOH was then added as the homogenization solution at a volume of 3 μL per mg of tissue. The samples were freeze-dried using nitrogen and stored at −80 °C until further processing. For homogenization, tissue samples were mechanically disrupted on ice using a Biomasher tissue homogenizer (Nippi, Tokyo, Japan), followed by vortexing for 10 s. We centrifuged samples at 18,341 × g at 4 °C for 10 min, and the supernatants were collected as tissue extracts and stored at −80 °C until further processing. To generate pooled quality control (PQC) samples, 20 µL of tissue extracts from each sample were combined into a single conical tube. For metabolite extraction, 50 µL of each tissue extract was transferred to a new 1.2-mL tube and mixed with 200 µL of MeOH: EtOH = 1:1 as an extraction solvent. After vortexing for 10 s, samples were centrifuged at 18,341 × g for 5 min at 4 °C. The final supernatants were transferred into two vials, each containing 100 µL, for untargeted metabolomics.

For lipid extraction, 20 μL of lung homogenate was mixed with 200 μL of 80% isopropanol, and 10 μL of internal standards (ISs) were added. The detailed compositions of the IS mixtures are listed in **Supplementary Table 1**. The mixtures were vortexed and centrifuged at 18,341 × g for 10 min at 4 °C. Subsequently, 180 μL of the supernatant was transferred into Eppendorf SafeLock tubes and subjected to liquid-liquid extraction based on a modified method described by Matyash et al. (21). Briefly, 600 μL of MTBE was added to each sample, followed by vortexing. Phase separation was induced by adding 180 μL of water, after which the mixture was vortexed again and centrifuged for 10 min. The mixture was vortexed again and centrifuged for 10 min. After centrifugation, 400 μL of the upper organic phase was collected and dried under nitrogen gas. The dried extracts were reconstituted with 200 μL of 95% ACN containing 10 mM AmAc.

### 2.6 Untargeted metabolomics

Samples were analyzed using an Orbitrap Exploris 120 Mass Spectrometer (Thermo Fisher Scientific, Bremen, Germany). An ACQUITY UPLC HSS T3, 1.8 µm 2.1 × 100 mm column (Waters Corporation, Milford, MA, USA) was connected to the ultra-performance liquid chromatography (UPLC) system. For the mobile phase, water containing 0.1% formic acid (mobile phase A) and MeOH with 0.1% formic acid (mobile phase B) were used under gradient conditions. Gradient elution started with 95% B at 0.5 min, decreased to 40% B at 6.5 min, and then to 40% B at 10 min. It increased to 95% B at 12 min and was maintained for 20 min. The flow rate was set at 0.25 mL/min from 0 to 12 min, increased to 0.4 mL/min from 13.0 to 18.0 min, and returned to 0.25 mL/min from 19 to 20 min. The column temperature was maintained at 40 °C. Analytes were detected with the following settings: positive ion, 3500 V; negative ion, 3500 V; sheath gas, 45 Arb; aux gas, 8 Arb; sweep gas, 1 Arb; ion transfer tube temperature, 350 °C; vaporizer temperature, 300 °C; resolution, 120000; RF lens, 35%; scan range, 67–800 m/z.

Raw data were processed using the Tidymass workflow in R (22). MS1 spectra were converted to the mzXML format and MS2 spectra to the mgf format using ProteoWizard (version 3.0.21256). For initial data preprocessing, the massprocesser package was used to perform peak detection, retention time correction, and feature alignment across samples. Subsequently, the massdataset package was used to construct an integrated data object containing feature-intensity matrices, sample metadata, and feature annotations. MS2 information from.mgf files was incorporated into the same object to facilitate downstream identification. Data cleaning was conducted using the masscleaner package to remove background noise and handle missing values. Metabolic features detected in at least 20% of QC samples and in at least 50% of samples within at least one experimental group were retained for further analysis. Metabolite annotation was performed using the MetID package by matching accurate mass, retention time, and MS2 fragmentation patterns against both public and in-house spectral libraries, including the Michael Snyder RPLC, Orbitrap, and MoNA databases. Compounds were annotated at confidence levels 1 and 2 according to the established identification criteria. Authentic reference standards were analyzed to confirm level 1 identifications, whereas level 2 identifications were assigned based on spectral similarity with library entries (23). The consistency of the compound annotation was validated through a combined evaluation of levels 1 and 2 identification results.

### 2.7 Transcriptomics

Lung tissues from the control and virus-infected mice were subjected to bulk RNA sequencing. Total RNA was extracted using TRIzol reagent, following the manufacturer’s instructions. Total RNA quality was assessed using the TapeStation 4000 System (Agilent Technologies, SantaClara, CA, USA). High-quality RNA samples were provided to Ebiogen (Seoul, South Korea) for library preparation and mRNA sequencing (mRNA-Seq). Libraries were constructed using the CORALL RNA-Seq V2 Library Prep Kit(Lexogen, Vienna, Austria) with Poly(A) selection and sequenced Illumina Stranded mRNA Prep Kit and sequenced on the Illumina NovaSeq 6000 platform (Illumina, San Diego, CA, USA). Adapter sequences and low-quality reads were trimmed using fastp. The quality-filtered reads were aligned to the mouse reference genome(GRCm39, NCBI) using STAR. Gene expression values were compared between groups using the Wilcoxon rank-sum test, and genes with an FDR < 0.05 were considered differentially expressed genes (DEGs).

### 2.8 Targeted lipidomics

Targeted lipidomic analysis was performed using a modified method of a previously developed hydrophilic interaction liquid chromatography-tandem mass spectrometry (HILIC-MS/MS) strategy (24). Lipidomic analysis was performed using an ACQUITY UPLC I-Class PLUS System coupled with a SCIEX Triple Quad 5500 Mass Spectrometer (Waters, Milford, MA, USA) operated in positive and negative ionization modes. Chromatographic separation was conducted on an ACQUITY Premier BEH Amide column (1.7 µm, 100 mm × 2.1 mm I.D., Waters, Milford, MA, USA) with a column temperature set at 40 °C and a flow rate of 0.3 mL/min. The injector needle was washed with ACN:water (90:10) after each injection and the mobile phase consisted of solvent A (ACN:water = 95:5) and solvent B (ACN:water = 50:50), both containing 10 mM AmAc. The gradient elution program was as follows: 0.1% B for 2 min, increased to 80% B over 2 min, returned to 0.1% B within 1 min, and equilibrated under the initial conditions for 3 min. Optimized ion source parameters included a voltage of 5500 V in positive mode and −4500 V in negative mode, an ion source temperature of 400 °C, and GS1 and GS2 settings of 30 and 35 psi, respectively. The curtain gas and collision-activated dissociation gas pressures were maintained at 20 and 6 psi, respectively. A scheduled multiple reaction monitoring (sMRM) algorithm with a retention time (tR) window of 1 min was used.

Data processing was conducted using Analyst version 1.6.3 (AB SCIEX), and the peak area of each lipid was normalized by dividing it by the peak area of the corresponding deuterated IS in the same lipid class. Correction for heavy isotopic overlap during lipid class-based chromatographic separation was performed using the Shiny application of LICAR, established by SLING (25). Identification of lipids using HILIC-MRM was achieved by determining the tR of lipid class-specific ISs. A total of four PQC samples were included to monitor instrument variability and ensure reliable lipid quantification. The filtering criteria were as follows: the relative standard deviations (RSDs) of the integrated peak area and peak height of the PQC samples were below 30%, and the tR RSD of the PQC samples was less than 5%.

### 2.9 Statistical analysis

In individual omics analyses, comparisons with controls are primarily conducted at 3 DPI to define acute-phase virus-specific molecular signatures at a common early infection time point. For SARS-CoV-2-infected mice, the pattern of body weight changes supports the validity of selecting this time point as the acute phase, with partial recovery by 6 DPI (**Supplementary Figure 1C**). In addition, a broader range of metabolic alterations was observed in infected mice at 3 DPI (**Supplementary Table 2**). For viral load, body weight changes, and lipid droplets, statistical significance was determined using the nonparametric Mann-Whitney U test.

For untargeted metabolomics, statistical analysis was performed using MetaboAnalyst 6.0 with log transformation and Pareto scaling (26). Metabolites that differed significantly between mice at 3 DPI and controls were identified based on statistical criteria. These metabolites were identified based on |log_2_ fold change (fc)| ≥ 1, FDR < 0.05. To statistically evaluate the group differences observed in the PCA plots, permutational multivariate analysis of variance (PERMANOVA) was performed using Euclidean distances, with 999 permutations.

For transcriptomics, statistical analyses were performed using the R software (version 4.4.1). Expression values for each gene were compared between groups using the Wilcoxon rank-sum test. For each gene, the log_2_ fold change was calculated as log_2_(mean expression in the 3 DPI-infected group/mean expression in control) using group-wise means with missing values removed. Genes with FDR <0.05 were considered significant and were exported for downstream analyses. Gene ontology (GO) terms were determined using g:Profiler (27). Integrated metabolomics and transcriptomics data were analyzed using the Joint Pathway Analysis module of MetaboAnalyst 6.0. Differentially expressed genes (DEGs) and significantly altered metabolites were mapped onto the Kyoto Encyclopedia of Genes and Genomes (KEGG) pathways to identify coordinated pathway changes. The integrated pathway results were visualized using R version 4.4.1 for customized graphical representation.

For targeted lipidomics, statistical analysis was performed using MetaboAnalyst 6.0 with log transformation and Pareto scaling. All data visualizations were generated using the R software (version 4.4.1). Upset plots were used to identify significantly altered lipids, and circular plots were used to compare log_2_ fold changes in overlapping lipid species between SARS-CoV-2 and MERS-CoV. Bubble plots showing lipid remodeling patterns by carbon chain length and degree of unsaturation were also produced in R (version 4.4.1), where bubble color indicated log_2_fc relative to controls and bubble size represented statistical significance (−log_10_*p*). Statistical significance was assessed using the non-parametric Wilcoxon rank-sum test for two-group comparisons.

### 2.10 Multi-omics integration with DIABLO

For multiomics integration, we used DIABLO, implemented in the mixOmics R package (28–30). DIABLO was applied after the single-omics analyses as a complementary integration step to identify coordinated cross-omics features that supported and refined the virus-specific signatures observed in the individual omics datasets. This supervised approach was selected because it maximizes covariance across omics layers while identifying correlated features that jointly contribute to discrimination among experimental groups. In this study, untargeted metabolomic, transcriptomic, and targeted lipidomic datasets obtained from mouse lung tissues (*n* = 4 per group) were integrated to identify the coordinated molecular signatures associated with infection. Before integration, each omics dataset was independently normalized and log transformed. Variables within each omics block were subsequently mean-centered and scaled to unit variance during DIABLO modeling to account for differences in measurement units and numerical ranges across the datasets. Features showing significant alterations between the experimental groups were selected as input variables for DIABLO analysis. The DIABLO model was fitted using the supervised block-sparse partial least squares discriminant analysis (block.splsda) algorithm, with the number of latent components set to two (ncomp = 2). The DIABLO model was constructed to discriminate among the three experimental groups: control, infected mice at 3 DPI, and infected mice at 6 DPI. The optimal number of variables selected in each omics block (keepX) was determined using the https://bioconductor.org/packages/release/bioc/html/mixOmics.html function with an M-fold cross-validation. The number of folds was adjusted according to the minimum sample size among the groups, with a maximum of three folds. The tuning procedure was repeated five times (nrepeat = 5). Classification performance was evaluated using the centroids-based distance (centroids.dist), and the optimal keepX combination derived from cross-validation was applied to the final block.splsda model. The latent components derived from the integrated model were visualized using score plots to evaluate group separation. Loading plots were generated to identify the top-ranked genes, metabolites, and lipids that contributed to group discrimination. Pairwise correlations among selected transcriptomic, metabolomic, and lipidomic features were calculated using Spearman correlation coefficients, and associations with an absolute correlation coefficient |*ρ*| ≥ 0.6 were retained for visualization. To evaluate whether the Spearman correlations were disproportionately influenced by any individual sample, a leave-one-out sensitivity analysis was performed (31, 32). Each of the 12 samples was omitted in turn, and Spearman’s correlation coefficient was recalculated using the remaining 11 samples. Directional stability was assessed by determining whether the sign of the correlation was retained across all leave-one-out iterations. In addition, the minimum absolute Spearman correlation coefficient across the 12 iterations was calculated as a descriptive measure of effect-size stability. To evaluate the influence of between-group separation on the pooled correlations, the corresponding group mean was subtracted from each feature value, and Spearman’s correlation coefficient was recalculated using the resulting residuals for the same feature pairs included in the original network (33). All DIABLO analyses and visualizations were performed using R (version 4.4.1).

**Figure 1:**
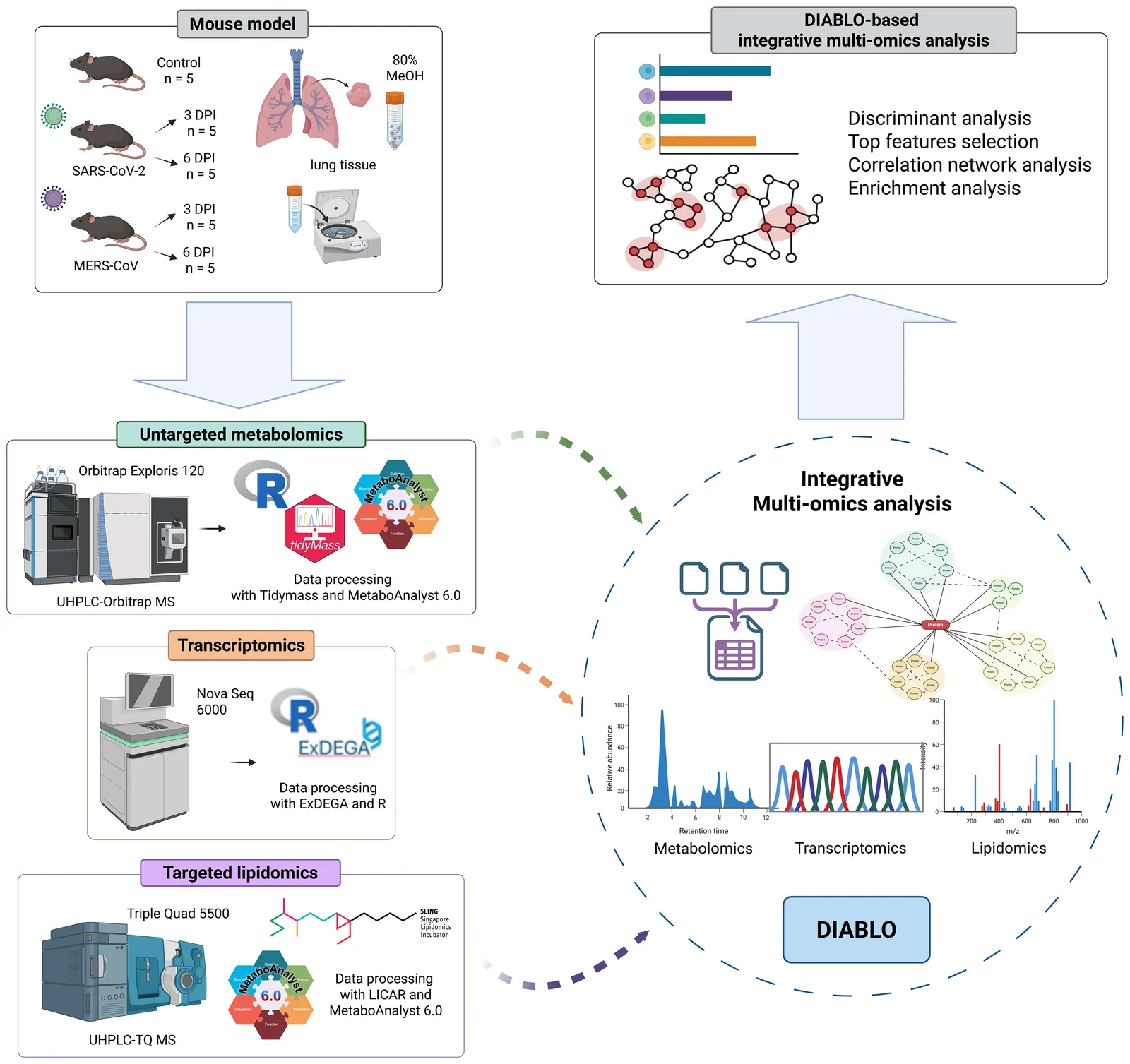
Schematic overview of the study design and DIABLO-based integrative multi-omics analysis. Created in BioRender. Snucpt, M. (2026) https://BioRender.com/cff5czi.

An integrated pathway analysis combining the three omics datasets was conducted using the Joint Pathway Analysis module in MetaboAnalyst 6.0 (34). DEGs and significantly altered metabolites and lipids were mapped onto KEGG pathways to identify coordinated changes in biological pathways. The significance of the enriched pathways was evaluated based on p-values and pathway impact scores, and bar plots were visualized using R (version 4.4.1). The overall workflow of the study and DIABLO-based integrative multi-omics analyses are summarized in **Figure 1**.

## 3 Results

### 3.1 SARS-CoV-2 and MERS-CoV induce distinct metabolomic profiles in infected mice lungs

**Figure 2:**
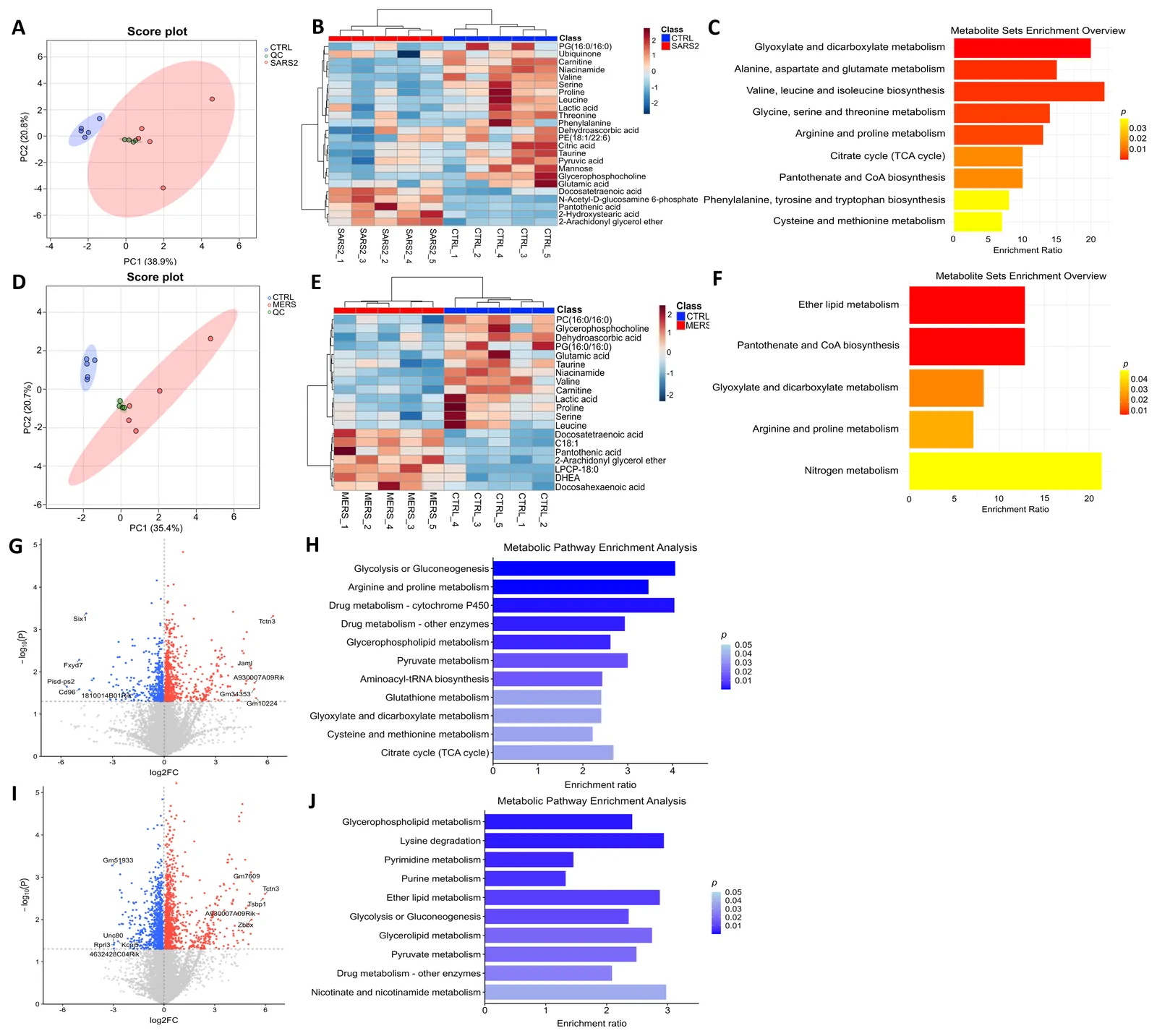
Multivariate analyses of untargeted metabolomics and transcriptomics in SARS-CoV-2 and MERS-CoV infected mouse at 3 DPI. **(A, D)** PCA score plots of metabolite profiles in SARS-CoV-2 **(A)** and MERS-CoV **(D)** versus controls. QC samples represent the pooled quality-control samples prepared from study samples and were included to monitor the analytical stability. **(B, E)** Heatmaps with hierarchical clustering of significantly altered metabolites in SARS-CoV-2 **(B)** and MERS-CoV **(E)**. **(C, F)** Pathway enrichment analysis of differential metabolites in SARS-CoV-2 **(C)** and MERS-CoV **(F)**. The color scale indicates pathway enrichment significance based on FDR-adjusted p-values. **(G, I)** Volcano plots of DEGs in SARS-CoV-2 **(G)** and MERS-CoV **(I)** (Wilcoxon rank-sum test, |fold change(fc)| ≥ 1, FDR < 0.05). **(H, J)** Joint pathway analysis integrating metabolomics and transcriptomics for SARS-CoV-2 **(H)** and MERS-CoV **(J)**. The color scale indicates pathway enrichment significance based on FDR-adjusted p-values.

To identify virus-specific metabolic changes at the tissue level, we performed untargeted liquid chromatography-mass spectrometry (LC–MS)-based metabolomics on lung tissues from hiACE2-hDPP4 DKI mice infected with SARS-CoV-2 or MERS-CoV at 3 DPI, along with uninfected controls (*n* = 5 per group). Principal component analysis (PCA) showed distinct clustering patterns between the infected and control groups for both SARS-CoV-2 (**Figure 2A**) and MERS-CoV (**Figure 2D**), indicating that both viruses substantially altered the lung metabolome. PERMANOVA with 999 permutations statistically supported the clustering patterns observed in the PCA, demonstrating significant differences between the infected and control groups for both SARS-CoV-2 (F = 3.194, *p* = 0.009) and MERS-CoV (F = 2.699, *p* = 0.009).

We identified 24 significantly altered metabolites in the SARS-CoV-2 group and 20 in the MERS-CoV group (**Table 1**; **Supplementary Figure 2**). Hierarchical clustering revealed that each virus induced a distinct set of upregulated and downregulated metabolites (**Figures 2B, E**). The pathway enrichment analysis revealed contrasting metabolic responses. In SARS-CoV-2-infected lungs, the most enriched pathways were related to amino acid metabolism, including glyoxylate-dicarboxylate, alanine–aspartate–glutamate, and arginine–proline metabolism as well as energy metabolism (**Figure 2C**). In the MERS-CoV-infected lungs, lipid-related pathways, including ether lipid metabolism, were dominant (**Figure 2F**).

**Table 1:** Summary of significantly changed metabolites identified by untargeted metabolomics in the two mouse infection models.

SARS-CoV-2 Metabolites	Fold Change	p value	MSI level
N-Acetyl-D-glucosamine 6-phosphate	2.77	0.008	2
2-Hydroxystearic acid	2.27	0.016	2
2-Arachidonyl glycerol ether	1.73	0.008	2
Docosatetraenoic acid	1.72	0.016	2
Pantothenic acid	1.47	0.008	1
Ubiquinone	0.73	0.032	2
Glutamic acid	0.70	0.032	1
Dehydroascorbic acid	0.69	0.016	2
Taurine	0.68	0.008	2
Pyruvic acid	0.66	0.008	2
Citric acid	0.61	0.016	2
Mannose	0.59	0.032	2
Phenylalanine	0.59	0.032	1
Threonine	0.58	0.008	1
Lactic acid	0.53	0.032	1
Proline	0.53	0.008	1
Leucine	0.47	0.008	1
Serine	0.46	0.008	1
Carnitine	0.43	0.008	2
PE(18:1/22:6)	0.43	0.032	2
Niacinamide	0.41	0.008	2
Valine	0.40	0.008	1
Glycerophosphocholine	0.34	0.008	2
PG(16:0/16:0)	0.30	0.032	2
MERS-CoV Metabolites	Fold Change	p value	MSI level
LysoPC(18:0)	2.43	0.016	2
DHEA	2.30	0.032	2
Docosatetraenoic acid	2.16	0.008	2
2-Arachidonyl glycerol ether	2.09	0.008	2
Docosahexaenoic acid	2.05	0.016	2
C18:1	1.94	0.008	2
Pantothenic acid	1.29	0.016	1
Serine	0.72	0.038	1
Taurine	0.68	0.008	2
Leucine	0.64	0.016	1
Dehydroascorbic acid	0.64	0.038	2
Proline	0.61	0.008	1
Lactic acid	0.61	0.008	1
Glutamic acid	0.58	0.008	1
Niacinamide	0.53	0.008	2
Carnitine	0.46	0.008	2
Glycerophosphocholine	0.46	0.032	2
PC(16:0/16:0)	0.42	0.008	2
Valine	0.40	0.008	1
PG(16:0/16:0)	0.30	0.032	2

Temporal analysis comparing 3 and 6 DPI showed that SARS-CoV-2-associated metabolite changes were partially attenuated by 6 DPI compared with those altered in MERS-CoV infection (**Supplementary Figure 2**).

### 3.2 Transcriptomic profiling reveals virus-specific immune and metabolic gene programs

Bulk RNA sequencing of lung tissues (*n* = 4 per group) at 3 DPI identified DEGs. SARS-CoV-2 infection resulted in 741 upregulated and 403 downregulated genes (**Figure 2G**), whereas MERS-CoV infection yielded 1,037 upregulated and 800 downregulated genes (**Figure 2I**).

KEGG pathway analysis of SARS-CoV-2 DEGs showed enrichment of regulation of actin cytoskeleton, virus infection, and phagosome pathways (**Supplementary Figure 3A**). GO analysis highlighted immune activation and cytoskeletal remodeling (**Supplementary Figure 3B**). For MERS-CoV, KEGG analysis identified the enrichment of virus infection, focal adhesion, and phagosome pathways (**Supplementary Figure 4A**), whereas GO analysis indicated viral process and cytokine production (**Supplementary Figure 4B**). Additionally, IFN-related genes were consistently upregulated in both SARS-CoV-2 and MERS-CoV infections at 3 and 6 DPI, with no significant downregulation (**Supplementary Figure 5**).

Integrated joint pathway analysis using MetaboAnalyst 6.0 showed that SARS-CoV-2-associated changes converged on glycolysis/gluconeogenesis and amino acid metabolism (**Figure 2H**), whereas MERS-CoV changes were concentrated on glycerophospholipid metabolism, lysine degradation, and purine/pyrimidine metabolism (**Figure 2J**). Network visualization revealed that relative to SARS-CoV-2, MERS-CoV displayed more prominent involvement in lipid and nucleotide metabolism. (**Supplementary Figures 3C**, **S4C**).

### 3.3 Targeted lipidomics reveals virus-specific lipid class remodeling at 3 DPI

**Figure 3:**
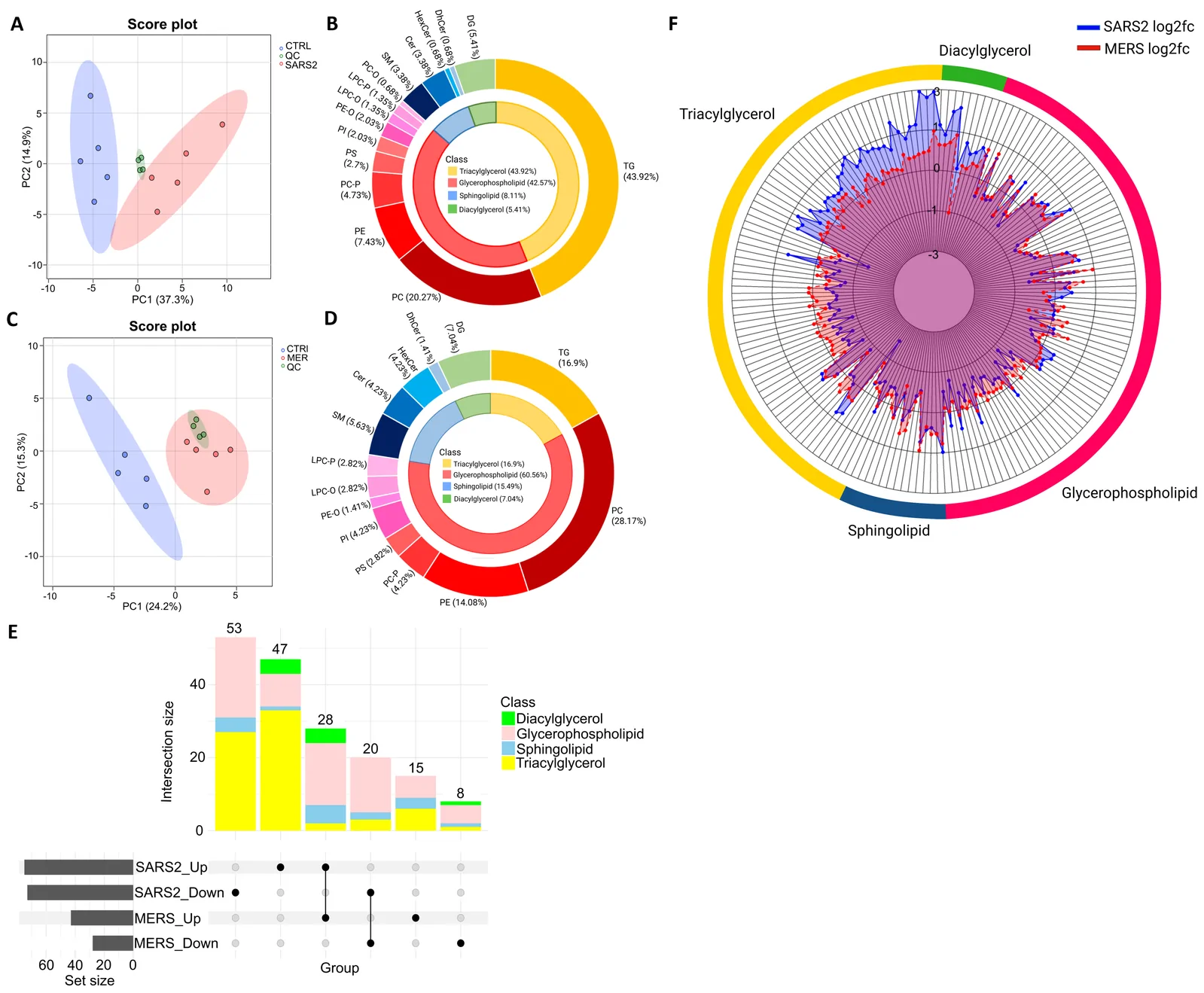
Targeted lipidomics at 3 DPI reveals virus-specific lipid class remodeling. **(A, C)** PCA score plots of lipid profiles in SARS-CoV-2 **(A)** and MERS-CoV **(C)** versus controls. **(B, D)** Donut plots showing lipid class composition of significantly altered species. **(E)** UpSet plot of shared and virus-specific lipid signatures. **(F)** Circular plot comparing log_2_ fold changes values relative to controls of overlapping lipid species that were commonly altered in both SARS-CoV-2 and MERS-CoV infection.

Targeted lipidomics, covering seven major lipid classes (TG, DG (diacylglycerol), PC (phosphatidylcholine), PE (phosphatidylethanolamine), LPC (lyso-phosphatidylcholine), SM (sphingomyelin), and Cer (ceramide)), was performed at 3 DPI (*n* = 5 per group). PCA confirmed distinct clustering patterns for both SARS-CoV-2 (**Figure 3A**) and MERS-CoV (**Figure 3C**). PERMANOVA with 999 permutations statistically supported the clustering patterns observed in the PCA, demonstrating significant differences between the infected and control groups for both SARS-CoV-2 (F = 3.573, *p* = 0.009) and MERS-CoV (F = 1.856, *p* = 0.004). In SARS-CoV-2-infected lungs, TG species accounted for the most significantly altered lipids (**Figure 3B**). In MERS-CoV-infected lungs, glycerophospholipids and sphingolipids accounted for a larger proportion of the altered lipids (**Figure 3D**).

The UpSet plot analysis demonstrated shared and virus-specific lipid signatures, showing that more lipid species were significantly altered by SARS-CoV-2 than by MERS-CoV at this time point (**Figure 3E**). Among the commonly altered lipid species, circular plot comparison of log_2_fc showed a prominent virus-specific divergence in neutral lipid remodeling, particularly within a subset of TG species (up to ~7.6) (**Figure 3F**). By comparison, MERS-CoV-associated lipids showed more modest changes.

### 3.4 Temporally divergent lipid remodeling dynamics between SARS-CoV-2 and MERS-CoV

**Figure 4:**
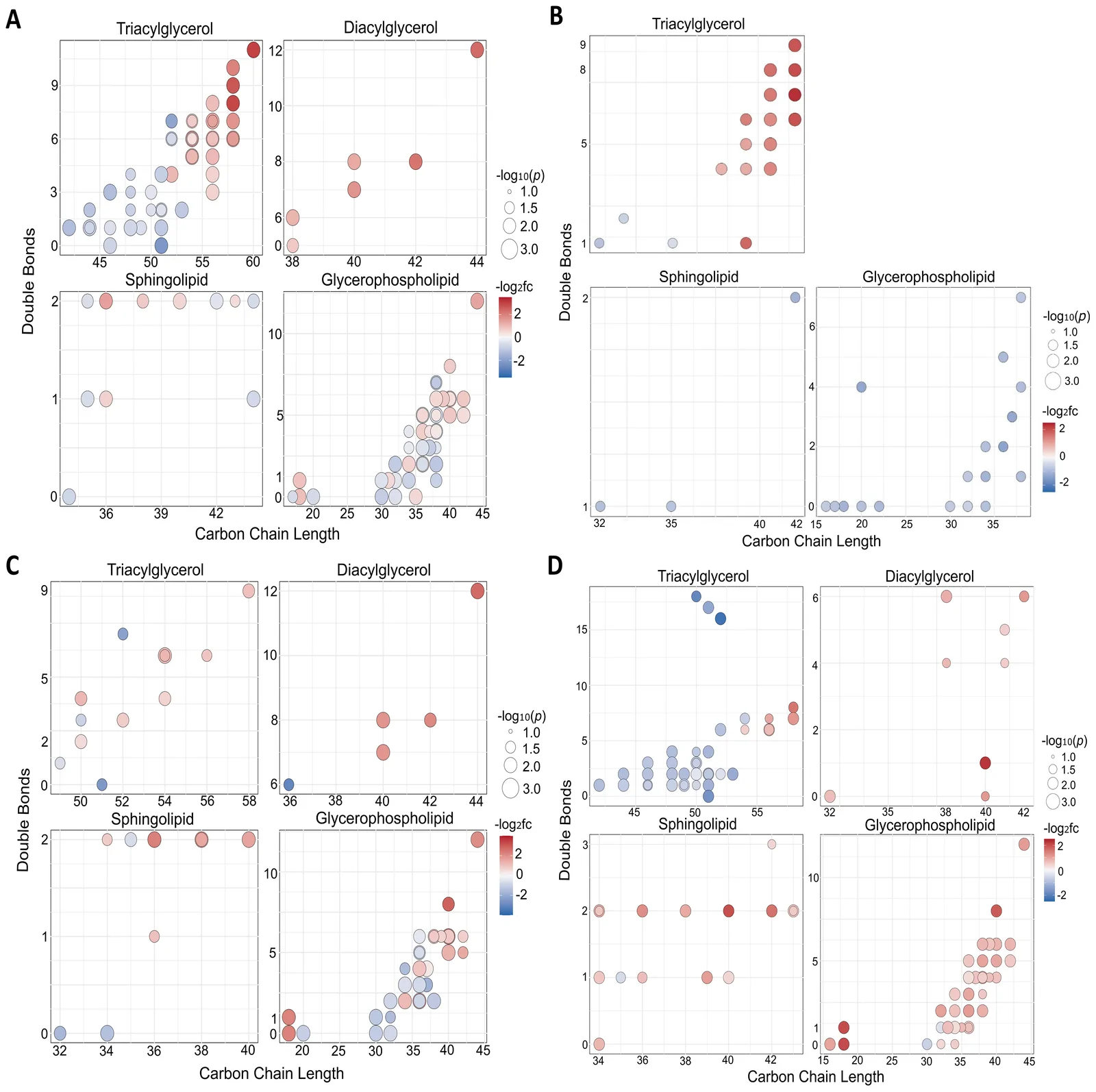
Temporally divergent lipid remodeling dynamics between SARS-CoV-2 and MERS-CoV infection. Bubble plots showing lipid changes stratified by carbon chain length and unsaturation degree. Color: log_2_fc; size: −log_10_*p*. **(A)** SARS-CoV-2, 3 DPI. **(B)** SARS-CoV-2, 6 DPI. **(C)** MERS-CoV, 3 DPI. **(D)** MERS-CoV, 6 DPI.

To further examine whether the lipids difference reflected broader temporal lipid remodeling, we analyzed lipid changes stratified by carbon chain length and degree of unsaturation at 3 and 6 DPI (**Figure 4**). SARS-CoV-2 showed pronounced lipid perturbations at 3 DPI, characterized by widespread increases in long-chain polyunsaturated TG and DG species (**Figure 4A**). By 6 DPI, many lipid species that were significantly altered in SARS-CoV-2-infected mice at 3 DPI shifted toward control-like levels (**Figure 4B**; **Supplementary Figure 6A**). MERS-CoV displayed the opposite pattern. At 3 DPI, lipid changes were modest (**Figure 4C**). MERS-CoV showed a delayed-progressive pattern, with lipid remodeling becoming more pronounced by 6 DPI and extending broadly across PC, PE, LPC, and sphingolipid species (**Figure 4D**; **Supplementary Figure 6B**). These contrasting kinetics indicate that the two viruses differ not only in the classes of lipids affected but also in the timing of lipid perturbation.

### 3.5 *In vitro* lipid droplet kinetics corroborate temporally divergent lipid utilization

**Figure 5:**
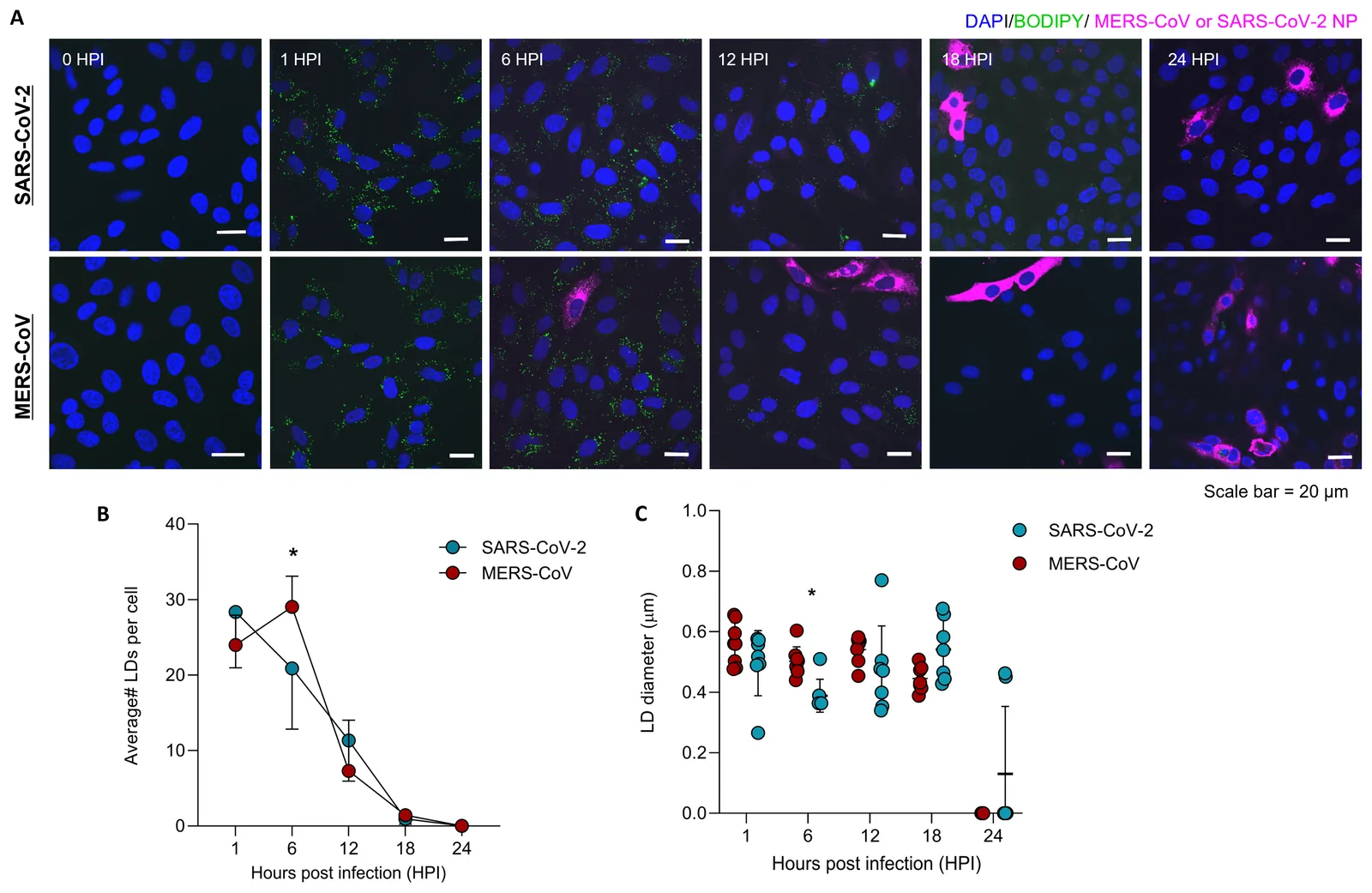
*In vitro* validation of temporally divergent lipid droplet dynamics. **(A)** Representative immunofluorescence images of SARS-CoV-2 and MERS-CoV infected Vero E6 cells stained with DAPI (blue), BODIPY (green), and anti-NP (red). Scale bar = 20 µm. **(B, C)** Quantification of the average number of lipid droplets per cell **(B)** and lipid droplet diameter **(C)** following infection with SARS-CoV-2 or MERS-CoV across the indicated time points. *p* value calculated by Mann-Whitney test. *p value < 0.05.

To confirm whether LD formation is specifically driven by active viral infection rather than non-specific cellular responses, we first compared cells treated with a solvent control, UV-inactivated virus, or live virus. LD accumulation was observed exclusively in live virus-infected cells, demonstrating that active viral infectivity is essential for LD biogenesis (**Supplementary Figure 7**). Based on these findings and to validate the *in vivo* findings, we examined LD dynamics in Vero E6 cells infected with SARS-CoV-2 or MERS-CoV at MOI 0.01. Cells were fixed at 1, 6, 12, 18, 24, 30, and 36 hours post-infection (HPI) and stained with BODIPY (lipid droplets), anti-NP antibodies (viral infection), and DAPI (nuclei) (**Figure 5A**; **Supplementary Figures 8A, B**).

Viral replication kinetics confirmed productive infections under both conditions (**Supplementary Figure 8C**). LD quantification showed that SARS-CoV-2-infected cells reached peak LD accumulation at 1 HPI, with a progressive decline thereafter, whereas MERS-CoV-infected cells peaked at 6 HPI (**Figure 5B**). At 6 HPI, MERS-CoV-infected cells displayed significantly increased LD numbers and diameters compared with SARS-CoV-2-infected cells (**Figures 5B, C**). SARS-CoV-2 showed an early increase in LDs, which gradually decreased over time, whereas MERS-CoV showed a later increase with larger LD accumulation. These results demonstrated that both SARS-CoV-2 and MERS-CoV infection induced LD accumulation in cultured cells, with distinct time-dependent patterns.

### 3.6 Multi-omics integration identifies coordinated TG–immune–metabolic signatures in SARS-CoV-2 infection

**Figure 6:**
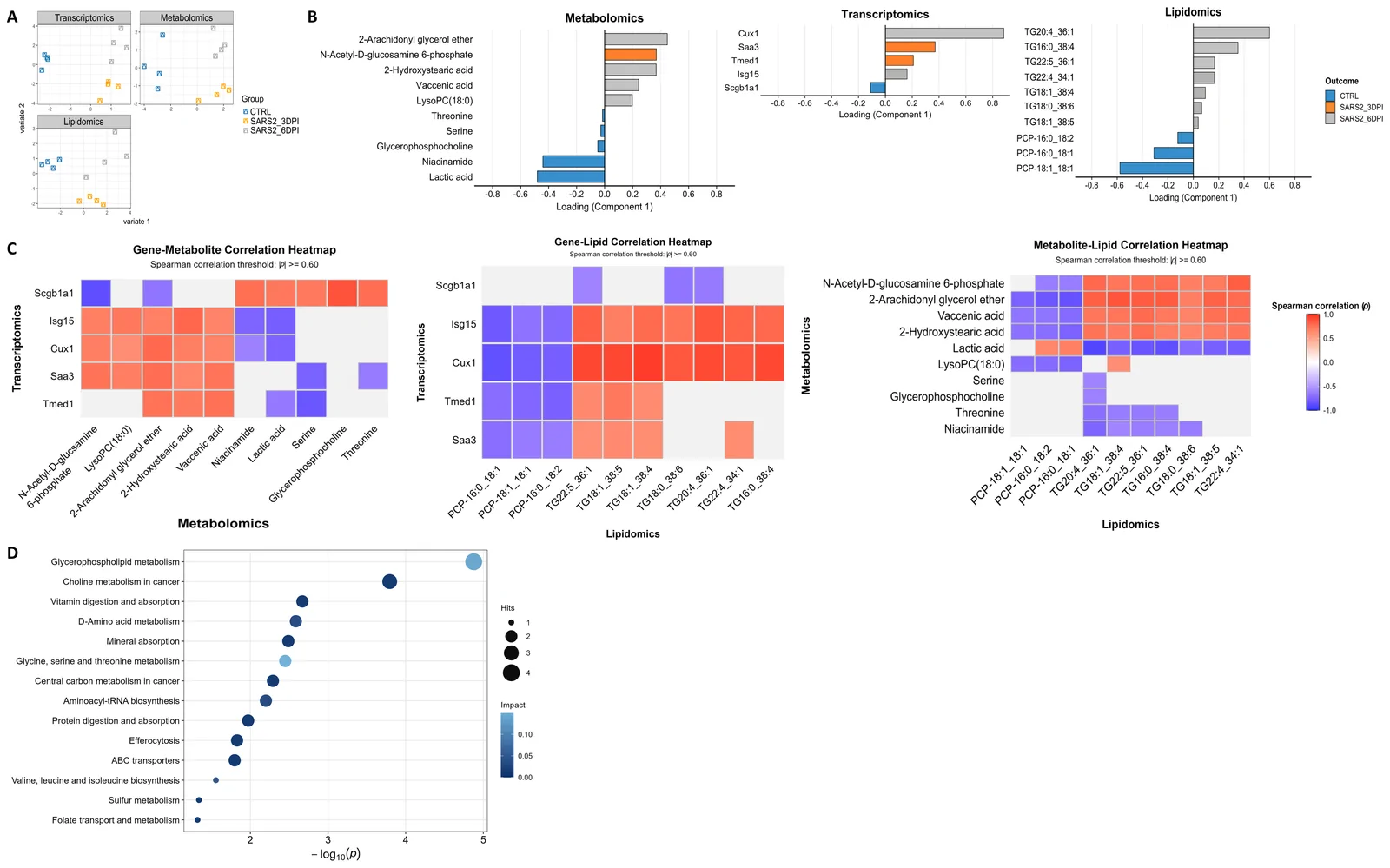
Integrative multi-omics analysis of SARS-CoV-2 infection. **(A)** DIABLO score plots show clear separation between 3 groups (control, SARS-CoV-2 infected mouse at 3 DPI and 6 DPI) based on the integrated multi-omics components. **(B)** Loading plots display the top-ranked metabolites, genes, and lipids contributing to discrimination among the control, 3 DPI, and 6 DPI groups along DIABLO Component 1. Each loading indicates the direction of the feature’s contribution to Component 1, whereas its absolute magnitude reflects the relative strength of that contribution. Bar colors indicate the group in which each feature showed the highest expression. **(C)** Pairwise Pearson correlation heatmaps showing associations between the selected transcriptomic, metabolomic, and lipidomic features during MERS-CoV infection. Gene–metabolite, gene–lipid, and metabolite–lipid correlations are displayed separately. Only strong correlations with an absolute Pearson correlation coefficient of |r| ≥ 0.70 are shown. Features that did not exhibit at least one correlation meeting this threshold with any feature in the paired omics layer were omitted entirely from the corresponding heatmap. Red indicates positive correlations, blue indicates negative correlations, and white cells indicate correlations that did not meet the predefined threshold (|r| < 0.70). **(D)** Pathway enrichment analysis of integrated multi-omics features. Circle size indicates the number of hits, and color represents the pathway impact.

To identify coordinated multiomics signatures, we applied the DIABLO framework to integrate untargeted metabolomics, transcriptomics, and targeted lipidomics into SARS-CoV-2-infected lung tissues. The block.splsda model, optimized by M-fold cross-validation, clearly separated the control, 3 DPI, and 6 DPI groups across all three omics layers (**Figure 6A**).

Loading plot analysis indicated the top-ranked features contributing to group discrimination along Component 1 (**Figure 6B**; **Supplementary Table 3**). 5 genes, 10 metabolites, and 10 lipids were selected for the model. Among the genes, Cux1 showed the highest positive loading (+0.88), followed by *Saa3* (+0.37), *Tmed1* (+0.21), and *Isg15* (+0.16), whereas *Scgb1a1* showed a negative loading (−0.11). Among metabolites, 2-arachidonyl glycerol ether (+0.45) and N-acetyl-D-glucosamine 6-phosphate (+0.37) were the top positive features, while lactic acid (−0.48) and niacinamide (−0.44) showed the strongest negative loadings. Among lipids, all seven selected TG species carried positive loadings (TG20:4_36:1, +0.60, being the highest), whereas all three plasmenyl-PC (PCP) species carried negative loadings (PCP-18:1_18:1, −0.58, being the most negative). This opposing directionality between the TG and PCP species indicates that TG accumulation and the lower relative levels of PCP are coordinately associated with the infection trajectory captured by the DIABLO model. Component 2 further distinguished the 3 and 6 DPI groups, with the temporal contrast being most evident in the transcriptomic block (**Supplementary Figure 9A**). However, features contributing to Component 2 exhibited fewer cross-omics correlations than those identified in Component 1 (**Supplementary Figure 9B**).

Spearman correlation network analysis (|*ρ*| ≥ 0.6, *p* < 0.05) of the DIABLO-selected variables revealed 132 significant inter-omics correlations among the 25 features (**Figure 6C**). The correlations were approximately balanced between the positive (56.8%) and negative (43.2%) associations. The expression level of immune- and infection-associated genes (*Isg15, Cux1*) were positively correlated with the levels of TG species (e.g., *Cux1*–TG18:1_38:4, *ρ* = +0.9580, *p* = 0.0001; *Isg15*–TG20:4_36:1, *ρ* = + 0.8671, *p* = 0.0017) but negatively correlated with the levels of lactic acid (e.g., *Isg15*–lactic acid, *ρ* = −0.790, *p* = 0.0025; *Cux1*–lactic acid, *ρ* = −0.769, *p* = 0.0023). Conversely, the expression level of lactic acid was negatively correlated with the levels of TG species (*ρ* = −0.72 to −0.91) and positively correlated with the levels of PCP species (*ρ* = +0.65 to +0.67). *Scgb1a1*, which showed an opposite loading direction, displayed corresponding inverse correlations: positive correlations with the levels of lactic acid and niacinamide (*ρ* = +0.72 and +0.76, respectively) and negative correlations with the levels of TG species. These inverse relationships indicate that glycolytic activity, TG accumulation, and *Isg15* expression, supported by immune-associated genes such as *Saa3, Tmed1*, and *Cux1*, represent temporally or mechanistically distinct facets of the SARS-CoV-2 host response rather than a single coordinately upregulated pathway. Glycerophospholipid, choline, and amino acid metabolism were identified as the most significantly enriched pathways (**Figure 6D**).

### 3.7 Multi-omics integration reveals a phospholipid–sphingolipid–membrane axis in MERS-CoV infection

**Figure 7:**
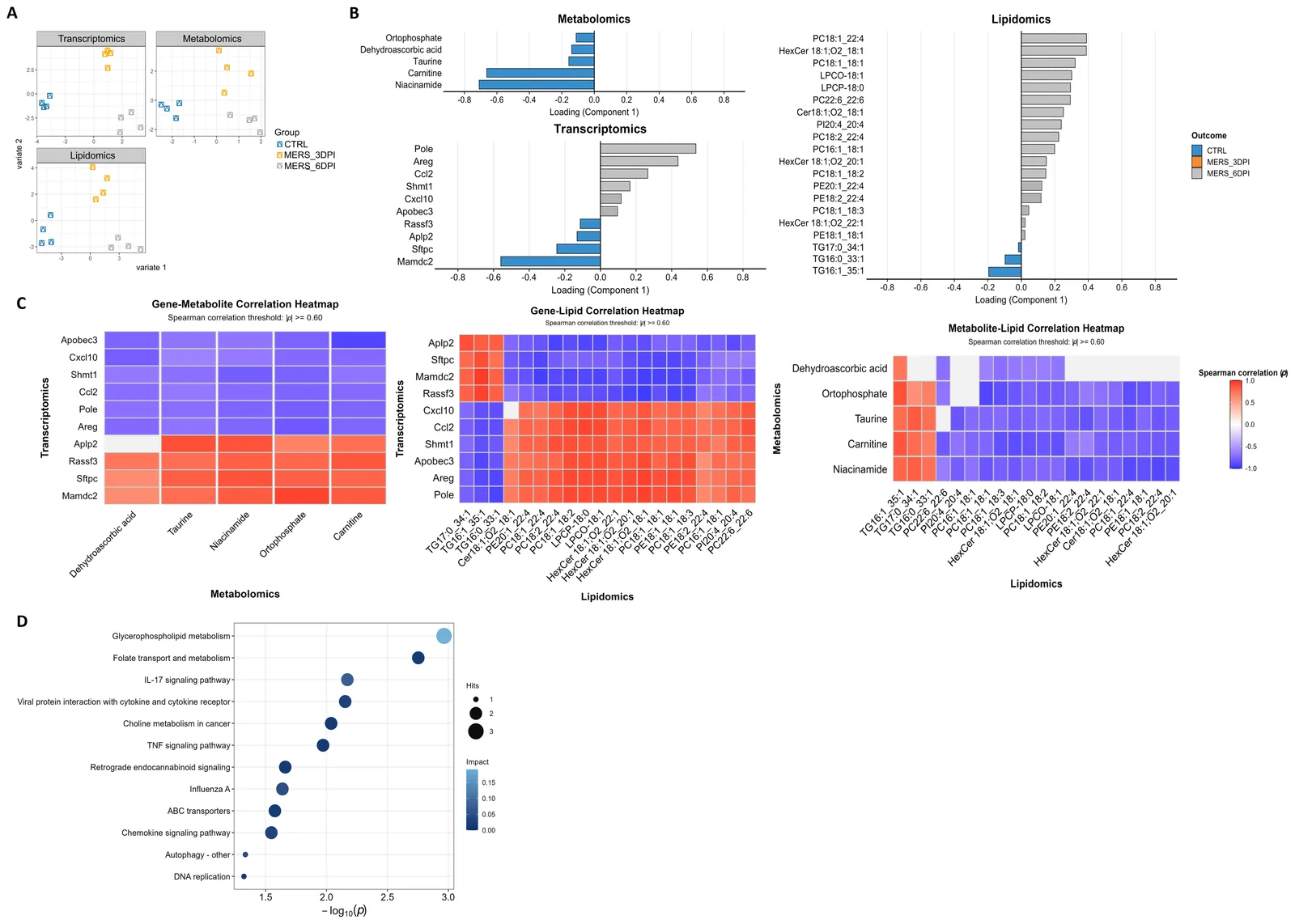
Integrative multi-omics analysis of MERS-CoV infection. **(A)** DIABLO score plots show clear separation between 3 groups (control, MERS-CoV infected mouse at 3 DPI and 6 DPI) based on the integrated multi-omics components. **(B)** Loading plots display the top-ranked metabolites, genes, and lipids contributing to discrimination among the control, 3 DPI, and 6 DPI groups along DIABLO Component 1. Each loading indicates the direction of the feature’s contribution to Component 1, whereas its absolute magnitude reflects the relative strength of that contribution. Bar colors indicate the group in which each feature showed the highest expression. **(C)** Pairwise Pearson correlation heatmaps showing associations between the selected transcriptomic, metabolomic, and lipidomic features during SARS-CoV-2 infection. Gene–metabolite, gene–lipid, and metabolite–lipid correlations are displayed separately. Only strong correlations with an absolute Pearson correlation coefficient of |r| ≥ 0.70 are shown. Features that did not exhibit at least one correlation meeting this threshold with any feature in the paired omics layer were omitted entirely from the corresponding heatmap. Red indicates positive correlations, blue indicates negative correlations, and white cells indicate correlations that did not meet the predefined threshold (|r| < 0.70). **(D)** Pathway enrichment analysis of integrated multi-omics features. Circle size indicates the number of hits, and color represents the pathway impact.

Parallel DIABLO analysis of MERS-CoV-infected lung tissues showed clear group separation across all three omics layers (**Figure 7A**). The model selected a larger and more diverse feature set than that of SARS-CoV-2: 10 genes, 5 metabolites, and 20 lipids, reflecting a broader molecular perturbation.

Loading plots indicated the top-ranked features driving group discrimination in Component 1 (**Figure 7B**; **Supplementary Table 4**). Among genes, two groups with opposing loadings emerged. Positive-loading genes included *Pole* (+0.54), *Areg* (+0.44), *Ccl2* (+0.27), *Shmt1* (+0.17), and *Cxcl10* (+0.12), indicating upregulation along the time-dependent response after infection; negative-loading genes included *Mamdc2* (−0.56) and *Sftpc* (−0.25), associated with lung epithelial injury. All five selected metabolites carried negative loadings, with niacinamide (−0.71) and carnitine (−0.66) showing the strongest, followed by taurine (−0.16), dehydroascorbic acid (−0.14), and orthophosphate (−0.11). Among the lipids, the selected set was markedly more diverse than that of SARS-CoV-2, comprising seven PC, three HexCer, three PE, two LPC, one PI, one Cer, and three TG species. Seventeen of the 20 lipid features, which were measured membrane-related lipid classes, carried positive loadings (PC18:1_22:4, +0.39, being the most positive), whereas the three TG species carried negative loadings (TG16:1_35:1, −0.19, being the most negative). This composition differed from the lipid profile of the SARS-CoV-2 group, in which 7 of the 10 selected lipids were TG species. Component 2 further distinguished the 3 and 6 DPI groups, with the temporal contrast being most evident in the transcriptomic and lipidomic blocks (**Supplementary Figure 8C**), with PC species becoming more prominent at 6 DPI. However, the Component 2 features exhibited a sparser correlation structure than those in Component 1 (**Supplementary Figure 8D**).

The correlation network analysis (**Figure 7C**) revealed 333 significant interomic correlations among the 35 features, with a higher proportion of negative correlations in the MERS-CoV network than in the SARS-CoV-2 network (56.4% vs. 43.2%). This pattern was driven primarily by metabolite–lipid associations, of which 72 of 85 edges (84.7%) were negative. In particular niacinamide and carnitine were inversely correlated with most membrane-associated phospholipid and sphingolipid species (e.g., niacinamide–PC18:1_22:4, *ρ* = − 0.9021, *p* = 0.0001; carnitine–HexCer 18:1;O2_18:1, *ρ* = − 0.8811, *p* = 0.0001). These correlations indicate an association between the levels of niacinamide and carnitine and those of the selected phospholipid and sphingolipid species across the groups.

Gene–lipid correlations revealed a clear functional division. The expression level of chemokine and infection-upregulated genes (*Ccl2, Cxcl10*) were strongly and positively correlated with the levels of membrane-related phospholipid and sphingolipid species (e.g., *Ccl2*–PC22:6_22:6, *ρ* = +0.8969, *p* = 0.0001; *Cxcl10*– PC18:1_18:2, *ρ* = +0.9041, *p* = 0.0002). The expression levels of inflammation related genes were correlated with the levels of the selected phospholipid and sphingolipid species. Conversely, genes with negative loadings (*Mamdc2, Sftpc*) showed the opposite pattern, with strong negative correlations to the same membrane lipid species (e.g., *Mamdc2*–PC18:1_22:4, *ρ* = −0.9441, *p* = 0.0001). The expression levels of *Mamdc2* and *Sftpc* were inversely correlated with those of the selected phospholipid and sphingolipid species across the groups. Pathway enrichment confirmed that glycerophospholipid metabolism, one-carbon metabolism, and cytokine signaling were the dominant coordinated pathways (**Figure 7D**).

Across the two viral infection models, leave-one-out sensitivity analyses indicated that the selected correlation networks were largely stable to the exclusion of individual samples. In the SARS-CoV-2 network, all 132 selected edges retained their original correlation direction, and 129 maintained |*ρ*| ≥ 0.5 across all iterations. In the MERS-CoV network, 312 of 333 selected edges retained their original direction, and 290 maintained |*ρ*| ≥ 0.5 throughout all iterations. To further assess whether the pooled correlations were influenced by separation among the experimental groups, Spearman correlations were recalculated after subtracting the corresponding group mean from each feature value. The subtraction of group means also reduced the available degrees of freedom, which was expected to attenuate residual correlations to some extent. The median absolute Spearman correlation decreased from 0.747 to 0.664 in the SARS-CoV-2 network and from 0.811 to 0.617 in the MERS-CoV network (**Supplementary Tables 5**, **S6**). Nevertheless, 102 of 132 SARS-CoV-2 edges (77.3%) and 226 of 333 MERS-CoV edges (67.9%) retained their original correlation direction, while 82 (62.1%) and 201 (60.4%) edges, respectively, maintained residual |*ρ*| ≥ 0.5 (**Supplementary Tables 5**, **S6**). These findings indicate that a substantial subset of the selected associations remained after adjustment for group mean effects.

Collectively, DIABLO integration and correlation network analyses revealed two distinct multiomics signatures that distinguished SARS-CoV-2 and MERS-CoV infections. These compositional and correlational differences in the DIABLO-selected networks were aligned with the divergent lipid utilization strategies identified by singleomics analyses: a TG-driven, temporally acute response in SARS-CoV-2 versus a broad membrane related lipid remodeling program in MERS-CoV.

## 4 Discussion

**Figure 8:**
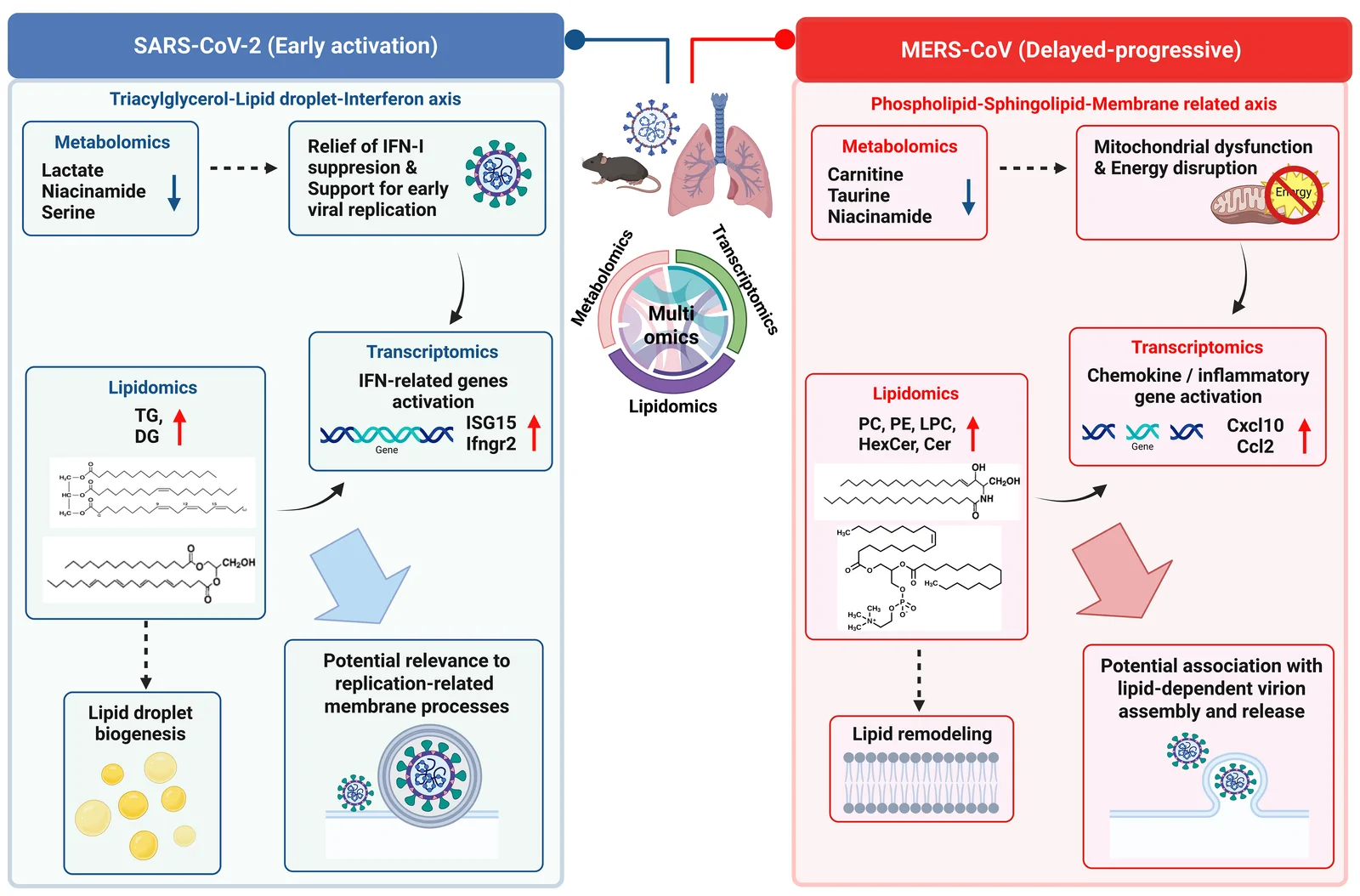
Schematic summary of the temporally and mechanistically distinct programs induced by SARS-CoV-2 and MERS-CoV infection. Created in BioRender. Snucpt, M. (2026) https://BioRender.com/gaqq9la.

The central finding of this study was that SARS-CoV-2 and MERS-CoV exploited host lipid metabolism through two fundamentally distinct, temporally programmed strategies (**Figure 8**). In SARS-CoV-2 infection, broad alterations across multiple lipid classes were observed at 3 DPI, whereas the lipid profile became more TG-dominant by 6 DPI. In contrast, MERS-CoV infection was characterized by progressively more pronounced alterations in phospholipid and sphingolipid species from 3 to 6 DPI. These lipid alterations were observed together with changes in inflammatory-related gene expression and decreases in energy-related metabolites.

These two strategies differed in the lipid classes involved (neutral lipids versus membrane lipids), temporal trajectory (early versus delayed-progressive), and transcriptional context (ISG–immune network versus chemokine–inflammatory network). We proposed that these differences reflect the fundamental ways in which each virus evolved to use the host cellular infrastructure. SARS-CoV-2 infection showed an earlier, relatively neutral lipid-dominant pattern associated with LD- and ISG-related features, whereas MERS-CoV infection showed delayed and sustained alterations in phospholipid and sphingolipid profiles associated with chemokine-related features. Below, we discuss the individual molecular features that constitute this framework and present our findings within the current literature.

The convergence of TG accumulation, LD biogenesis, and ISG activation positioned the TG–LD axis as the central metabolic feature of SARS-CoV-2 infection. DIABLO integration identified long-chain polyunsaturated TG species as the dominant lipid feature (7 of 10 selected lipids), with strong positive correlations with immune-related genes such as *Isg15* (*r* = +0.89) and *Cux1* (*r* up to +0.94). Dias et al. have demonstrated that LDs fuel SARS-CoV-2 replication and the production of inflammatory mediators (17), whereas Monson et al. have shown that early LD accumulation is required for efficient IFN responses (18). Recent studies have supported this hypothesis. Liu et al. have demonstrated that the coronaviral nsp6 directly binds to LD membranes, hijacks the endoplasmic reticulum-associated protein degradation (ERAD) machinery to degrade perilipin-2, and promotes lipolysis (35). The released fatty acids were directly supplied through the expansion of double-membrane vesicles (DMVs), and ERAD-derived vesicles provide essential membrane components to DMVs (35). Consistent with this, Farley et al. established that LD plasticity is a point of SARS-CoV-2 infection across variants (36). They have also shown fatty acid synthase (FASN) upregulation and lysosomal acid lipase (LIPA) downregulation, suggesting increased fatty acid production and reduced lipid breakdown, which may promote lipid accumulation inside cells (36). We observed that SARS-CoV-2 induced broader lipid metabolic changes than MERS-CoV at 3 DPI *in vivo*, together with earlier LD accumulation *in vitro*. These findings are consistent with the nsp6-mediated LD-to-DMV lipid flux model and suggest that SARS-CoV-2 rapidly mobilizes LD-derived lipids to support DMV membrane biogenesis during early infection. Our data suggest that this axis is temporally dynamic in SARS-CoV-2, peaking at 3 DPI and being partially resolved by 6 DPI. The *in vitro* experiments demonstrated that both SARS-CoV-2 and MERS-CoV infections induced LD accumulation, although the time points at which LD accumulation was most pronounced differed between the two viruses. In this context, LD biogenesis may be interpreted as an indicator of the temporal onset of broader lipid metabolic activation.

Evidence supports the bidirectional regulation of type I IFN signaling and lipid metabolism. York et al. have shown that limiting cholesterol biosynthetic flux spontaneously engages type I IFN signaling (37). Blanc et al. have demonstrated the IFN-mediated downregulation of sterol biosynthesis (38), and Wu et al. established that type I IFNs remodel core metabolic pathways, including fatty acid oxidation and glycolysis (39). In our SARS-CoV-2 DIABLO network, *Isg15* was positively correlated with TG species (*r* = +0.68 to +0.86) while being inversely correlated with lactic acid (*r* = −0.79) and niacinamide (*r* = −0.88). *Isg15* was not among the DIABLO-selected genes in MERS-CoV, suggesting that the ISG–lipid connection is a more prominent feature of SARS-CoV-2 infection. This observation is consistent with a model in which IFN-associated lipid remodeling promotes LD formation, which serves as both an antiviral signaling platform and a substrate for viral replication (18).

DIABLO analysis identified lactic acid and niacinamide as the metabolites with the strongest negative loadings (−0.48 and −0.44, respectively) in SARS-CoV-2 infection, indicating their depletion during infection. Previous studies have shown that lactate metabolism can modulate antiviral innate immune signaling; for example, Zhang et al. demonstrated that lactate directly inhibits RLR-MAVS-mediated type I IFN signaling at the molecular level (40), and other viral studies have reported that lactate accumulation suppresses antiviral innate immunity (41). These studies provide an association for the observed correlation between decreased lactic acid levels and IFN-related features in the SARS-CoV-2 DIABLO network. These findings suggest that lactate depletion may be linked to IFN-related antiviral responses in the context of infection-associated metabolic remodeling. However, because reduced food intake and body weight loss during infection may also influence systemic energy metabolism, lactate depletion may additionally reflect indirect effects of altered nutrient availability and increased energy demand. Although serine was selected by DIABLO, its loading was close to zero (−0.03). This suggests that it contributes to group discrimination primarily through interomics correlations rather than as a strong individual discriminator. Nevertheless, the concurrent decrease in serine levels and the increase in ISG expression were notable findings. Shen et al. have reported that serine restriction enhances IFN-β production and antiviral responses (42), raising the possibility that serine depletion during SARS-CoV-2 infection may amplify the type I IFN response.

In contrast to the TG-dominated SARS-CoV-2 signature, MERS-CoV infection was characterized by progressively more pronounced alterations in phospholipid and sphingolipid profiles. DIABLO selected a markedly more diverse lipid profile: seven PC, three HexCer, three PE, two LPC, one PI, and one ceramide species (17 of 20 lipids with positive loadings), accompanied by strong positive correlations with the chemokine genes *Ccl2* (e.g., *Ccl2*–PC22:6_22:6, *ρ* = +0.90) and *Cxcl10* (e.g., *Cxcl10*– PC18:1_18:2, *ρ* = +0.90). The pattern was more pronounced at 6 DPI than at 3 DPI, with broader and more marked alterations in PC, PE, LPC, and sphingolipid species. The functional significance of membrane related lipid accumulation has been reported previously. Zandi et al. provided a comprehensive framework for understanding how different coronaviruses differentially exploit lipid metabolism (43). Critically, Soultsioti et al. have demonstrated that in MERS-CoV-infected cells, the inhibition of *de novo* lipogenesis markedly reduced virion particle release without affecting DMV formation or viral RNA replication (44). Exogenous palmitic acid supplementation partially rescued viral activity, and the authors suggested that spike and envelope protein palmitoylation was an essential lipid-dependent step (44). These findings provide a broader biological context for considering the potential relevance of lipid metabolic alterations during MERS-CoV infection. These lipid changes may be associated with lipid-dependent processes previously implicated in virion assembly and viral particle release. Although we did not directly determine the functional roles of individual lipid species, the progressively altered phospholipid and sphingolipid profiles identify candidate lipid signatures that may be relevant to these lipid-dependent processes and provide a basis for future mechanistic studies.

Three metabolites (carnitine, taurine, and niacinamide) were commonly identified as top-ranked DIABLO features in MERS-CoV (loadings: −0.66, −0.16, and −0.71, respectively), all showing negative loadings indicative of depletion during infection. Carnitine is essential for mitochondrial fatty acid import via the carnitine shuttle (45). Taurine is critical for mitochondrial tRNA modification and respiratory chain function (46). Niacinamide is the precursor of NAD^+^, a central cofactor in cellular energy metabolism (47), and Heer et al. have demonstrated that coronavirus infection and poly(ADP-ribose) polymerase hyperactivation deplete cellular NAD^^+^^ pools (47). The coordinated depletion of all three metabolites during MERS-CoV infection suggests a broad disruption of mitochondrial energy homeostasis. Specifically, carnitine depletion indicates impaired fatty acid β-oxidation, taurine depletion points to compromised mitochondrial translation accuracy, and niacinamide consumption reflects reduced NAD^^+^^ availability. However, these changes may also be influenced by reduced food intake during infection. In the DIABLO correlation network, these metabolites showed strong inverse correlations with levels of membrane-related lipid species (e.g., niacinamide–PC18:1_22:4, *ρ* = −0.90; carnitine–HexCer 18:1;O2_18:1, *ρ* = −0.88), suggesting an association between lower metabolite levels and higher levels of these membrane-related lipid species. Importantly, our mouse model findings are consistent with those of human COVID-19 metabolomic studies. Shen et al. have reported widespread metabolic dysregulation in the serum of patients with COVID-19, including amino acid and lipid alterations (11). Song et al. demonstrated systems-level metabolic dysregulation using multiomics (48), and Thomas et al. reported increased serum glucose and free fatty acids, together with decreased lactate and short- and medium-chain acylcarnitines in patients with COVID-19 (12). In our study, lactate was similarly decreased in SARS-CoV-2-infected mouse lungs. Free carnitine was also decreased, showing a similar direction of change to the decreases in acylcarnitines reported by Thomas et al. However, while the human serum study primarily identified alterations in circulating free fatty acids, our data showed changes in long-chain polyunsaturated lipids at 3 DPI. Thus, both studies indicated infection-associated disruptions of energy and lipid metabolism. These observations placed our mouse lung tissue findings within the broader metabolic alterations reported in human COVID-19 while also highlighting tissue- and model-specific differences.

The identification of temporally programmed, virus-specific lipid utilization strategies has direct implications for host-directed antiviral therapy design. For SARS-CoV-2, the therapeutic window has appeared earlier. Targeting TG synthesis (diacylglycerol O-acyltransferase 1 (DGAT1) inhibitors) during the initial days of infection, when the TG–LD axis is most active, may maximally disrupt viral replication. This approach was supported by Williams et al., who have shown that DGAT1 inhibition reduce SARS-CoV-2 replication (49), and Farley et al., who have demonstrated that TG accumulation is a conserved feature across SARS-CoV-2 variants (36). In our MERS-CoV model, alterations in phospholipid and sphingolipid profiles became progressively more pronounced from 3 to 6 DPI. LD accumulation was most pronounced at the later measured time point following MERS-CoV infection under the experimental conditions used. Previous studies have shown that fatty acid synthase inhibition selectively blocks MERS-CoV virion release without affecting replication organelle formation and that fatty acid synthase is linked to lipid droplet biogenesis (44, 50). Together, these observations raise the possibility that lipid-dependent host processes, including lipid droplet metabolism, are associated with lipid metabolic alterations accompanying MERS-CoV infection. Further studies are needed to determine their contribution to infection and to define the therapeutic potential and optimal timing of lipid-targeted interventions.

There were some limitations in this study. First, although the similarity between mouse and human metabolomic signatures supports translational relevance, species differences in immune responses and receptor distribution may affect the strength and timing of these metabolic changes, so direct extrapolation to human disease should be interpreted with caution. Second, the sample size (*n = 5* per group), which is commonly used in exploratory animal multi-omics studies (51, 52), limits the statistical power; therefore, future studies with larger cohorts would strengthen confidence in the identified signatures. Third, although our *in vitro* LD data showed temporal trends consistent with the *in vivo* findings, the differences did not reach statistical significance at all time points. This may be partly attributable to the impaired interferon responses in Vero cells (53); therefore, further validation under higher MOI conditions and in additional cell systems is warranted. In addition, SARS-CoV-2 and MERS-CoV utilize distinct entry receptors, ACE2 and DPP4, respectively, resulting in different cell-type tropisms. These differences in target cell populations may contribute to distinct host immune responses, metabolic reprogramming, and lipidomic profiles. Therefore, future studies incorporating cell-type–resolved multi-omics approaches are needed to better define the mechanisms driving these virus-specific host responses. Fourth, lung tissues were collected without vascular perfusion prior to snap-freezing to minimize ex vivo metabolic alterations associated with prolonged tissue handling. Therefore, residual intravascular blood may have influenced a subset of the measured metabolites and transcripts. However, because all samples were harvested using an identical standardized protocol with minimal ischemic time, this potential contribution is expected to be consistent across experimental groups. Fifth, as the individual food intake was not directly monitored, the potential contribution of infection-associated reductions in food consumption to the observed host metabolic changes cannot be excluded. Finally, the two-timepoint design (3 and 6 DPI) provided limited temporal resolution. While the correlation results support coordinated cross-omics associations among genes, lipids, and metabolites, they should be interpreted with caution regarding causal directionality or direct mechanistic interpretations between these molecular layers due to the limited number of post-infection time points. Future studies with larger cohorts, more detailed time-course designs, and improved *in vitro* validation will help strengthen and refine the proposed mechanistic model.

## 5 Conclusions

This study provides the first direct, three multiomics comparison of SARS-CoV-2 and MERS-CoV in hiACE2-hDPP4 DKI mice. We established that the two β-CoVs exploit the host lipid metabolism through temporally programmed, distinct and temporally programmed host lipid remodeling patterns. SARS-CoV-2 infection was characterized by broad early increases in TG and DG species, together with LD accumulation and IFN-related transcriptional features, with many of the lipid alterations partially attenuated by 6 DPI. In contrast, MERS-CoV infection showed delayed and progressively more pronounced alterations in phospholipid and sphingolipid profiles at 6 DPI. As suggested by previous studies, these alterations may be associated with lipid-dependent viral processes, such as virion assembly and release. Decreased tissue lactate levels were suggested to be associated with IFN-related features in the SARS-CoV-2 multi-omics network, suggesting a possible relationship between altered energy metabolism and antiviral immune responses. These findings provide a mechanistic framework for understanding the pathogenesis of different coronaviruses and for identifying temporally defined therapeutic nodes for virus-specific, host-directed antiviral strategies.

## Data Availability

The datasets presented in this study can be found in online repositories. The names of the repository/repositories and accession number(s) can be found below: https://kbds.re.kr, KAP242354.

## Glossary

DKI: double knock-in
DIABLO: Data Integration Analysis and Biomarker discovery using Latent cOmponents
DPI: Days post infection
TG: Triacylglycerol
DG: Diacylglycerol
PC: Phosphatidylcholine
PE: Phosphatidylethanolamine
LPC: Lysophosphatidylcholine
PI: Phosphatidylinositol
PCP: plasmenyl-PC
SM: Sphingomyelin
CER: Hexcer Hexosylceramide
LD: Lipid droplet
IFN: Interferon
ISG: Interferon-stimulated genes
ARDS: Acute respiratory distress syndrome
ACE2: Angiotensin-converting enzyme 2
DDP4: Dipeptidyl peptidase 4
MeOH: Methanol
ACN: Acetronitrile
EtOH: Ethanol
MTBE: Methyl tert-butyl ether
AmAc: Ammonium Acetate
QC: Quality control
IS: Internal standard
LLE: Liquid-liquid extraction
UPLC: Ultra-performance liquid chromatography
HILIC-MS/MS: hydrophilic interaction liquid chromatography-tandem mass spectrometry
sMRM: scheduled Multiple Reaction Monitoring
tR: Retention time
PQC: Pooled quality control
RSDs: Relative standard deviations
fc: fold change
DEGs: Differentially expressed genes
GO: Gene ontology
KEGG: Kyoto Encyclopedia of Genes and Genomes
HPI: hours post-infection
NP: Nucleoprotein
DAPI: 4′,6-diamidino-2-phenylindole
PLIN2: Perilipin-2
DMVs: Double-membrane vesicles
ERAD: Endoplasmic-Reticulum-Associated Protein Degradation
RLR-MAVS: RIG-I-Like Receptor-Mitochondrial Antiviral-Signaling Protein
PARP: Poly (ADP-ribose) polymerase
DGAT: Diacylglycerol O-acyltransferase
FASN: Fatty Acid Synthase
LIPA: Lipase A
MOI: Multiplicity of Infection.
