# Supplementary material for: Divergent lipid utilization strategies of SARS-CoV-2 and MERS-CoV revealed by comparative multi-omics profiling of infected mouse lung tissues
Source: Front Immunol. 2026 Aug 25;17:1902981. doi: 10.3389/fimmu.2026.1902981 (PMC13550176; doi:10.3389/fimmu.2026.1902981)
Supplement: Supplementary file 1 [file DataSheet1.pdf]

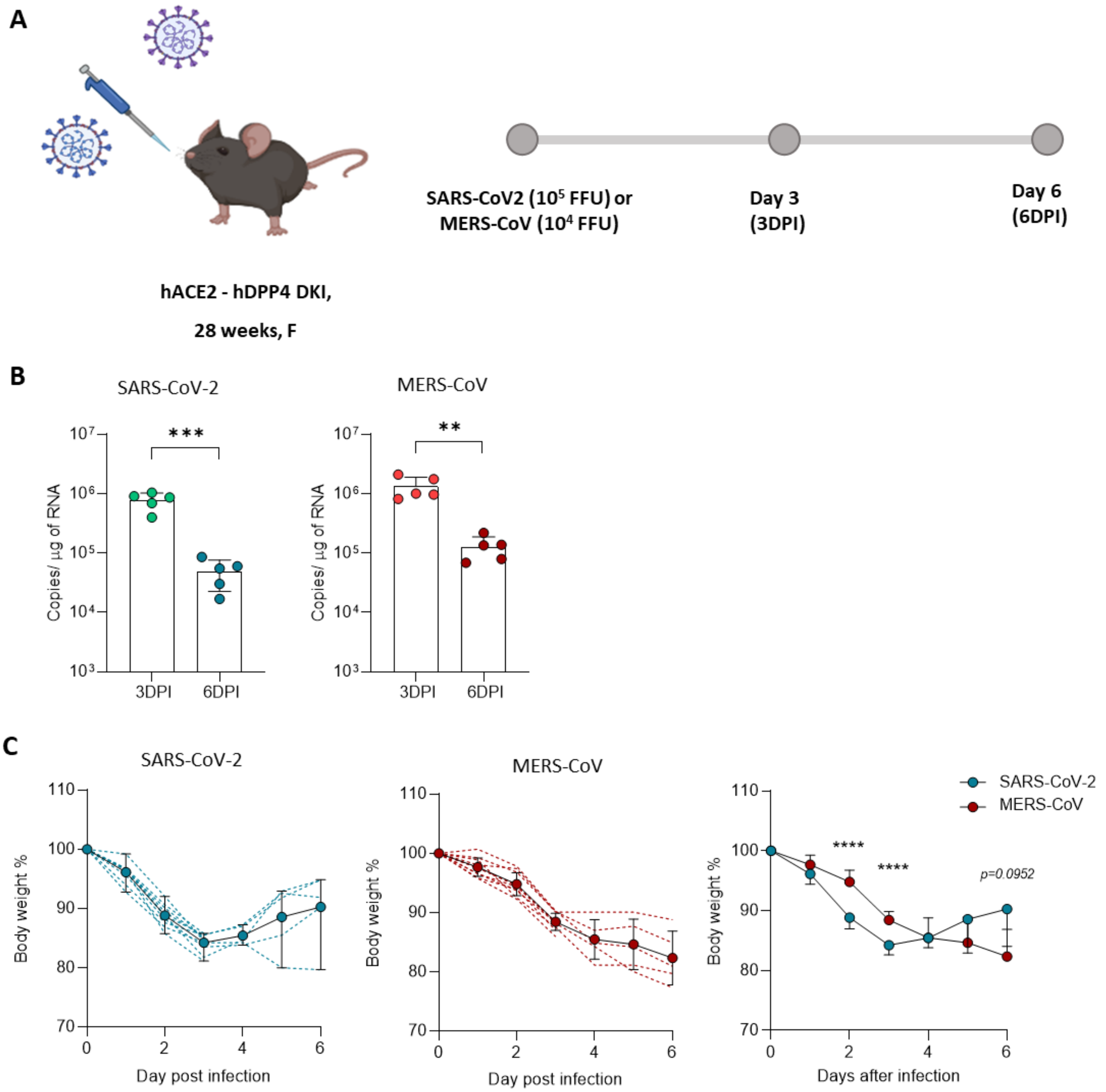

Figure S1. Animal model schematic (A), viral load (B), and body weight changes (C) of infection mouse

(A, B) Animal model shematic and viral load in mouse lung at 3, 6 DPI. *p* value calculated by the Nonparametric Mann-Whitney test.

(C) Body weight changes of hiACE2-hDPP4 mice infected with 10^5 FFU of the SARS-CoV-2 and 10^4 FFU of MERS-CoV. *p* value calculated by the Nonparametric Mann-Whitney test.

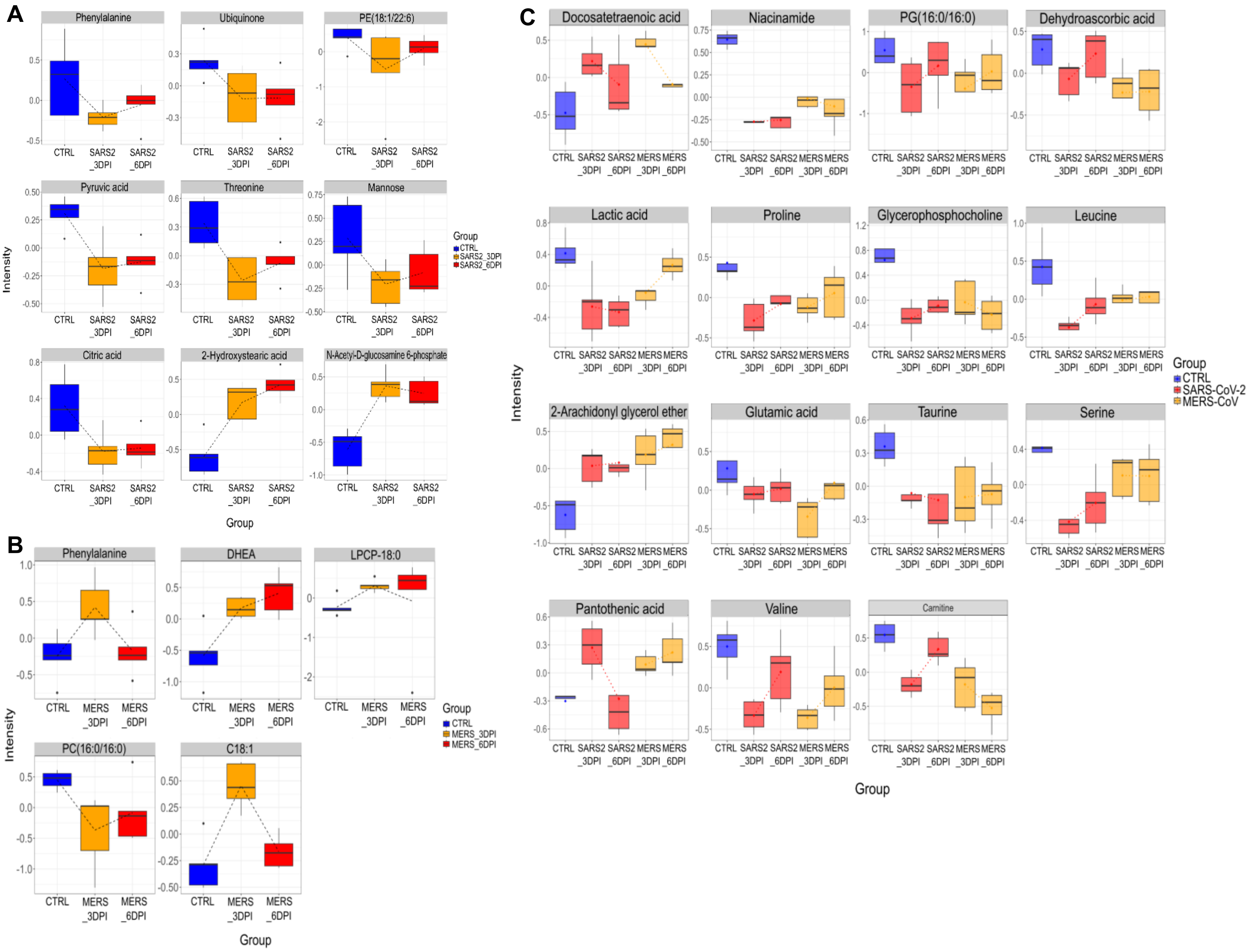

Figure S2. Temporal changes of significant metabolites revealed by untargeted metabolomics.

(A, B) Box plots showing temporal changes of significant metabolites that were specifically altered in SARS-CoV–2 3DPI mouse (A) and MERS-CoV 3DPI mouse (B) compared with controls.

(C) Box plots showing temporal changes of significant metabolites that overlapped between SARS-CoV-2 and MERS-CoV infection.

Metabolites shown were pre-selected based on statistical significance from comparisons between infected and control groups (3 DPI vs. CTRL) using a Wilcoxon rank-sum test.

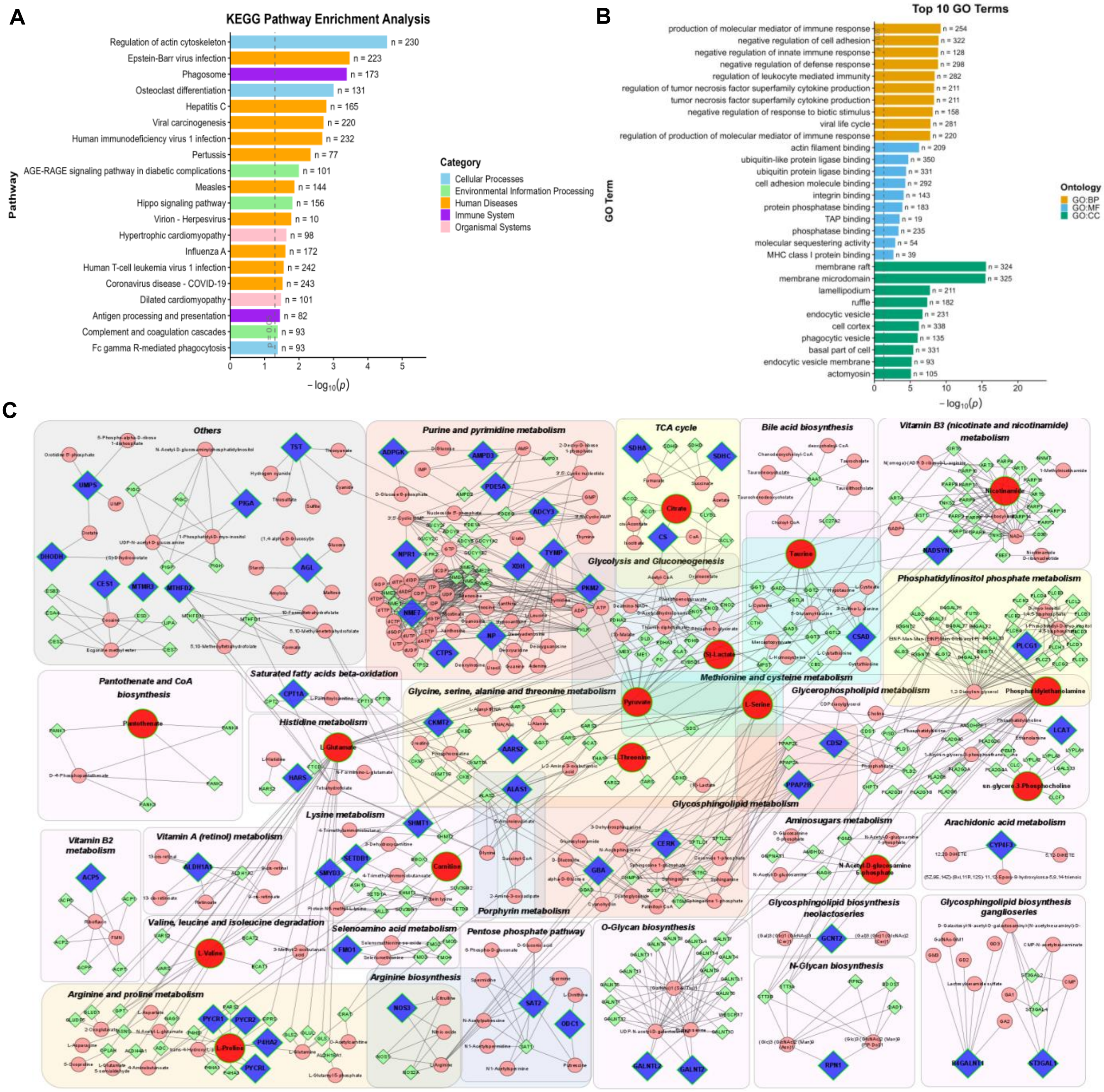

Figure S3. Transcriptomics-based functional enrichment analysis and integrated metabolite–gene networks in SARS-CoV-2–infected mouse.

(A) KEGG pathway enrichment analysis based on the differentially expressed genes. Bar lengths represent the $-\log_{10}$-FDR-adjusted p-values, with larger values indicating greater statistical significance. Bar colors indicate the corresponding KEGG pathway categories. The numbers adjacent to the bars indicate the gene-set size (n) associated with each pathway.

(B) Gene Ontology (GO) enrichment analysis showing the top 10 significantly enriched terms within each ontology: biological process (BP), molecular function (MF), and cellular component (CC). Bar lengths represent the $-\log_{10}$-FDR-adjusted p-values, and bar colors indicate the GO ontology. The numbers adjacent to the bars indicate the gene-set size (n) associated with each GO term. The

dashed vertical line in panels A and B indicates the significance threshold corresponding to an FDR-adjusted p-value of 0.05.

(C) Integrated network analysis combining transcriptomic and untargeted metabolomic data in SARS-CoV-2–infected mouse. Diamond-shaped nodes represent genes and circular nodes represent metabolites. Blue diamonds indicate significantly altered genes, and red circles indicate significantly altered metabolites.

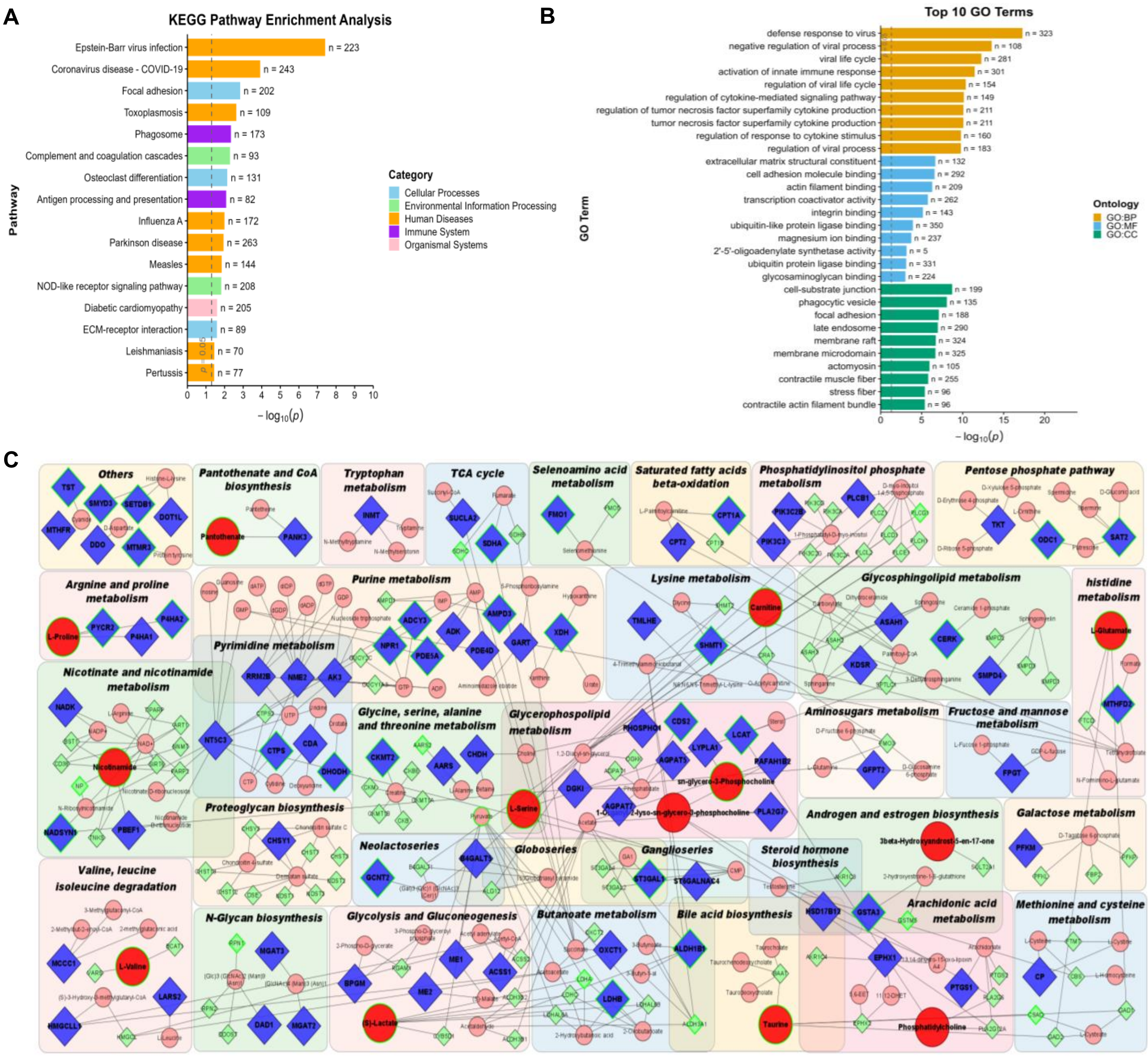

Figure S4. Transcriptomics-based functional enrichment analysis and integrated metabolite–gene networks in MERS-CoV–infected mouse.

(A) KEGG pathway enrichment analysis based on the differentially expressed genes. Bar lengths represent the $-\log_{10}$-FDR-adjusted p-values, with larger values indicating greater statistical significance. Bar colors indicate the corresponding KEGG pathway categories. The numbers adjacent to the bars indicate the gene-set size (n) associated with each pathway.

(B) Gene Ontology (GO) enrichment analysis showing the top 10 significantly enriched terms within each ontology: biological process (BP), molecular function (MF), and cellular component (CC). Bar lengths represent the $-\log_{10}$-FDR-adjusted p-values, and bar colors indicate the GO ontology. The numbers adjacent to the bars indicate the gene-set size (n) associated with each GO term. The

dashed vertical line in panels A and B indicates the significance threshold corresponding to an FDR-adjusted p-value of 0.05.

(C) Integrated network analysis combining transcriptomic and untargeted metabolomic data in MERS-CoV–infected mouse. Diamond-shaped nodes represent genes and circular nodes represent metabolites. Blue diamonds indicate significantly altered genes, and red circles indicate significantly altered metabolites.

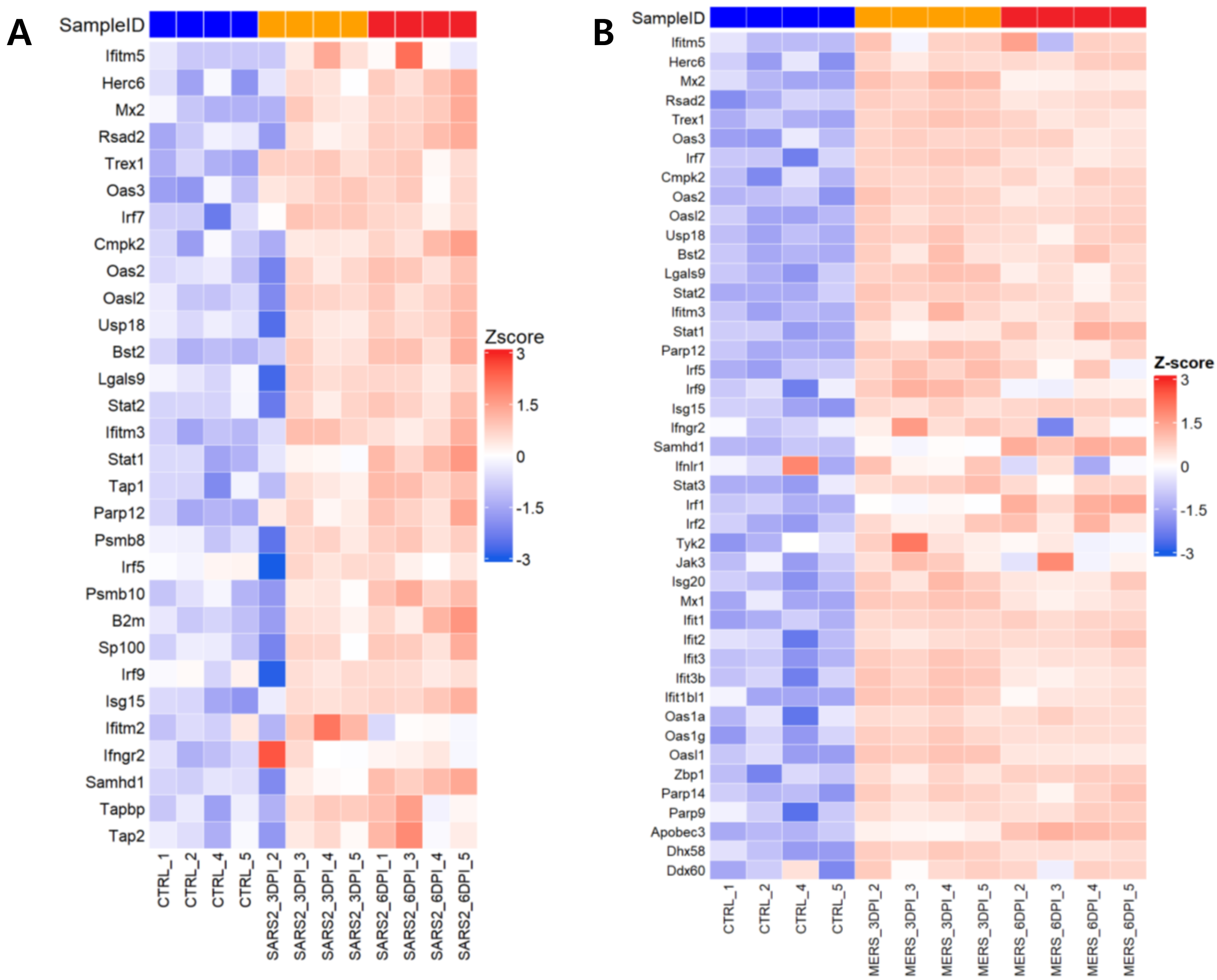

Figure S5. Temporal clustering of significantly altered IFN-related genes in SARS-CoV-2 3 DPI and MERS-CoV 3 DPI infected mice.

(A, B) Heatmaps showing hierarchical clustering of significantly upregulated IFN-related genes (Cluster 1) in SARS-CoV-2–infected mice (A) and MERS-CoV–infected mice (B) across control, 3 DPI, and 6 DPI. No significantly downregulated IFN-related genes were identified. Gene expression levels are presented as Z-scores.

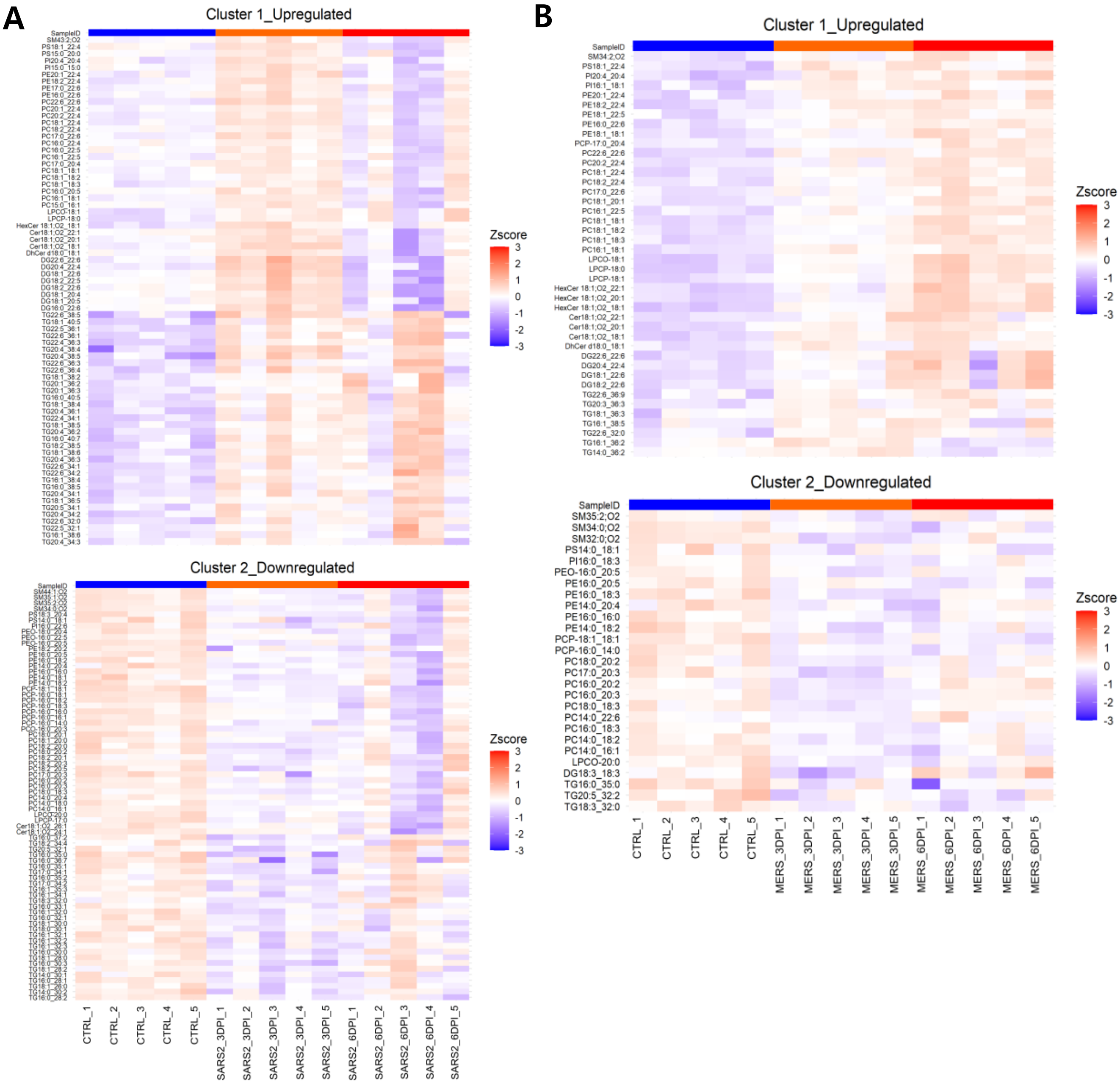

Figure S6. Temporal clustering of significantly altered lipids in SARS-CoV-2 3 DPI and MERS-CoV 3 DPI infected mice.

(A, B) Heatmaps showing hierarchical clustering of significantly upregulated (Cluster 1) and downregulated (Cluster 2) lipid species in SARS-CoV-2 infected mice (A) and MERS-CoV infected mice (B) across controls, 3DPI, and 6DPI. Lipid intensities are shown as Z-scores.

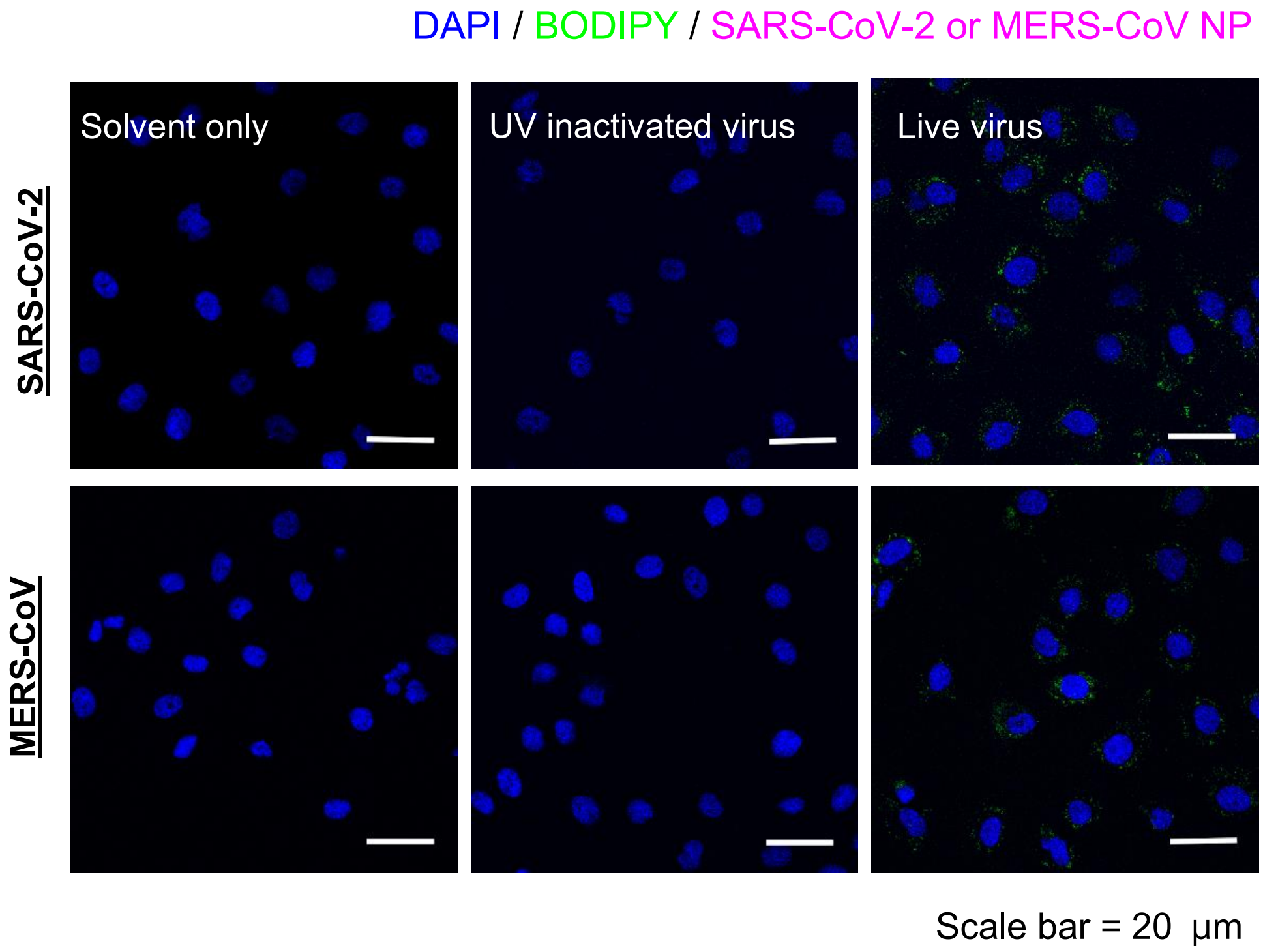

Figure S7. Comparison of lipid droplet formation in response to live and UV-inactivated virus.

Immunofluorescence analysis of lipid droplet accumulation in Vero E6 cells at 1hpi following infection with live or UV-inactivated SARS-CoV-2 and MERS-CoV, or treatment with solvent control. Nuclei and lipid droplets are stained with DAPI(blue) and BODIPY(green), respectively.

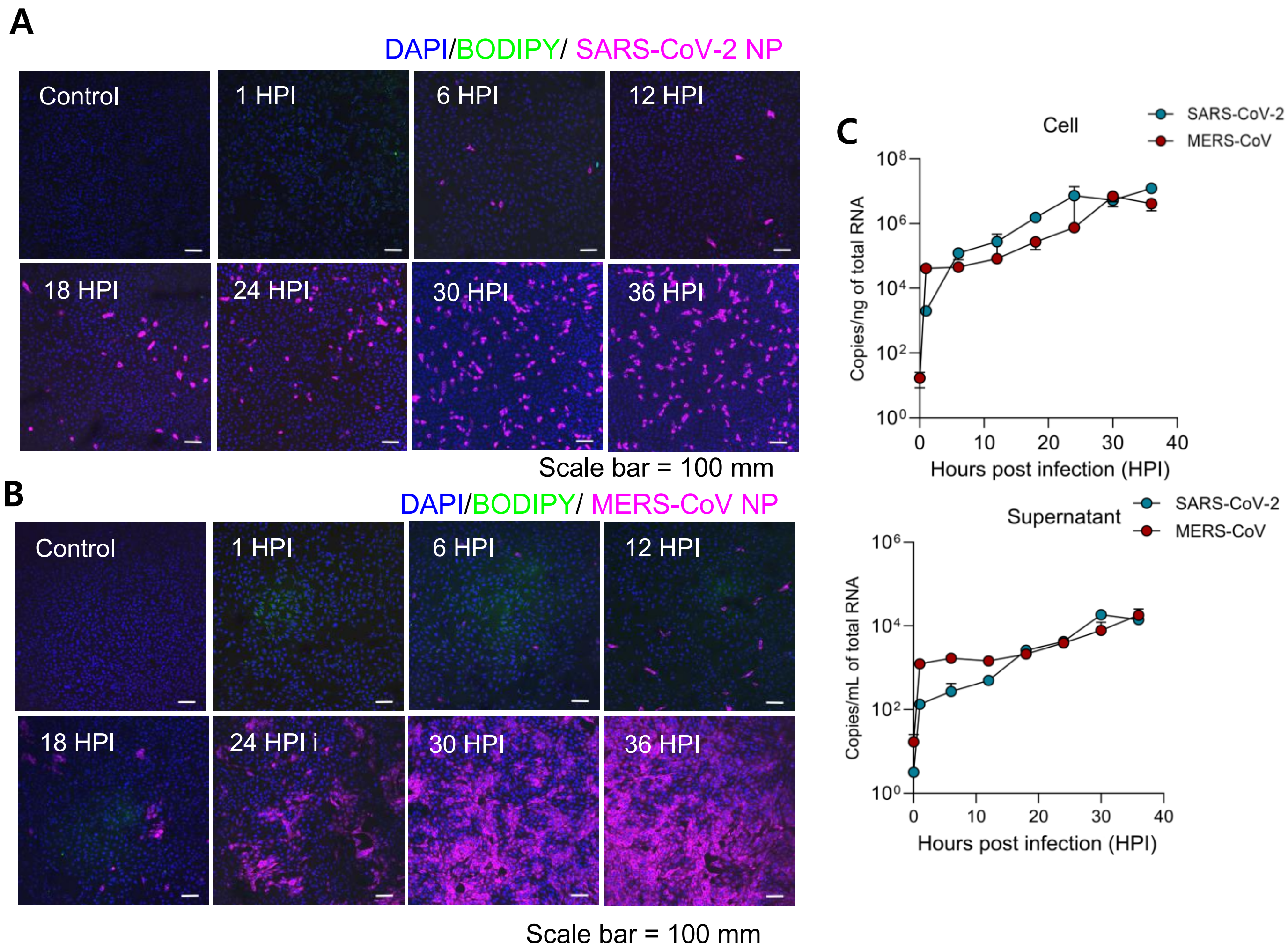

Figure S8. Lipid droplet formation in SARS-CoV-2- and MERS-CoV-infected Vero E6 cells.

(A, B) Immunofluorescence images showing lipid droplet formation in Vero E6 cells infected with SARS-CoV-2 (A) or MERS-CoV (B) at the indicated time points (control, 1, 6, 12, 18, 24, 30 and 36 hpi). Nuclei are stained with DAPI (blue), lipid droplets are labeled with BODIPY (green), and infected cells are detected by viral nucleoprotein (NP) staining (magenta).

(C) Viral replication kinetics in Vero E6 cells infected with SARS-CoV-2 or MERS-CoV. Intracellular viral RNA levels are expressed as copies per ng of total cellular RNA (copies/ng), while extracellular viral RNA levels measured in culture supernatants are expressed as copies per milliliter (copies/mL).

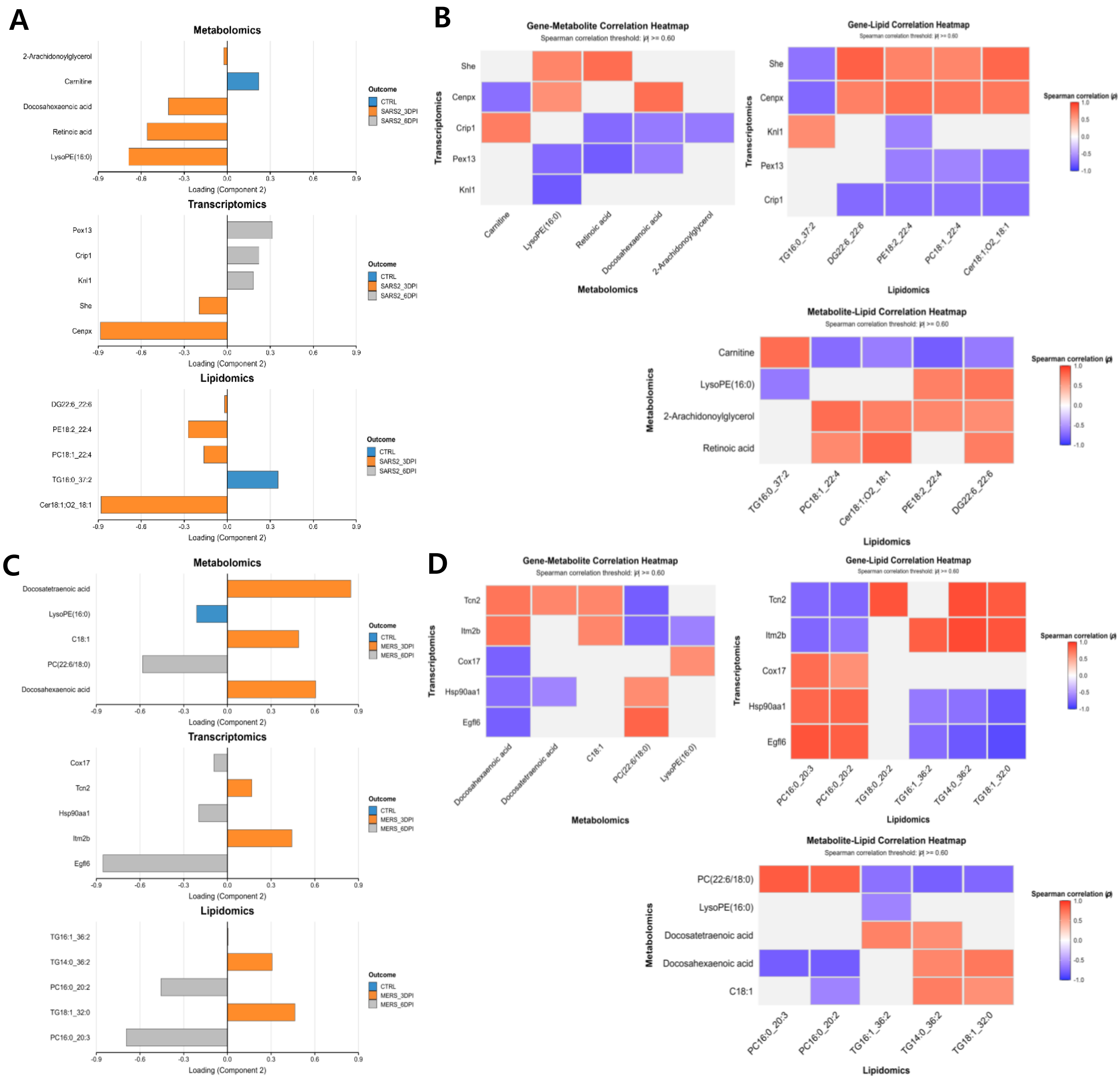

Figure S9. Loading plots and Pairwise Pearson correlation heatmaps of DIABLO component 2.

(A, C) Loading plots display the top-ranked features contributing to discrimination among the three groups along DIABLO component 2 of SARS-CoV-2 (A) and MERS-CoV (C) infection mice.

(B, D) Sperman correlation heatmaps showing associations between the selected transcriptomic, metabolomic, and lipidomic features during SARS-CoV-2 (B) and MERS-CoV (D) infection. Only strong correlations with an absolute Sperman correlation coefficient of $|\rho| \geq 0.6$ are shown. Features that did not exhibit at least one correlation meeting this threshold with any feature in the paired omics layer were omitted entirely from the corresponding heatmap. Red indicates positive correlations, blue indicates negative correlations, and white cells indicate correlations that did not meet the predefined threshold ($|\rho| < 0.6$).

Table S1. The detailed composition of the internal standard (IS) mixture of targeted lipidomics

Standard	Source	Represents	Conc. Stock (ng/mL)	Diluted in	Initial Conc (ng/mL)	Final Conc (ng/mL)
15:0-18:1(d7) PC160	330707	PC	160	Methanol	160	4000
15:0-18:1(d7) PE5	330707	PE	5	Methanol	5	125
15:0-18:1(d7) PG30	330707	PG	30	Methanol	30	750
18:1(d7) LPC25	330707	LPC	25	Methanol	25	625
18:1(d7) LPE5	330707	LPE	5	Methanol	5	125
15:0-18:1(d7) DG10	330707	DG	10	Methanol	10	250
15:0-18:1(d7)-15:0 TG55	330707	TG	55	Methanol	55	1375
18:1(d9) SM30	330707	SM	30	Methanol	30	750
15:0-18:1(d7) PA7	330707	PA	7	Methanol	7	175
15:0-18:1(d7) PS	330707	PS	5	Methanol	5	125
15:0-18:1(d7)PI	330707	PI	10	Methanol	10	250
CE18:1-d7	330707	CE	350	Methanol	350	8750
AC2:0 (d3)	Individual (Sigma)	AC	50	MTBE	5	125
Cer16:0-d7	Individual (Sigma)	Cer	50	MTBE	5	125
FA16:0-d31	Individual (Sigma)	FA	50	MTBE	2.5	62.5
HexCer 18:1-d7;O2_15:0	Individual (Sigma)	HexCer	50	MTBE	10	250
DhCer 18:1-d7;O2_13:0	Individual (Sigma)	DhCer	50	MTBE	5	125
Hex2Cer 18:1-d7;O2_15:0	Individual (Sigma)	Hex2Cer	50	MTBE	10	250

Table S2. Summary of differential metabolites identified by pairwise comparisons between controls and SARS-CoV-2- or MERS-CoV-infected mice at 3 and 6 DPI.

SARS2 3DPI vs CTRL		SARS2 6DPI vs CTRL		MERS 3DPI vs CTRL		MERS 6DPI vs CTRL	
**Metabolites**	**Up/Down**	**Metabolites**	**Up/Down**	**Metabolites**	**Up/Down**	**Metabolites**	**Up/Down**
Docosatetraenoic acid	↑	2-Hydroxystearic acid	↑	LysoPC(18:0)	↑	DHEA	↑
Pantothenic acid	↑	Pyruvic acid	↓	DHEA	↑	2-Arachidonylglycerol ether	↑
Ubiquinone	↓	N-Acetyl-D-glucosamine6-phosphate	↑	Docosatetraenoic acid	↑	Pantothenic acid	↑
Glutamic acid	↓	Threonine	↓	2-Arachidonyl glycerol ether	↑	Taurine	↓
Dehydroascorbic acid	↓	Citric acid	↓	Docosahexaenoic acid	↑	Niacinamide	↓
Taurine	↓	2-Arachidonyl glycerol ether	↑	C18:1	↑	Glycerophosphocholine	↓
Mannose	↓	Proline	↓	Pantothenic acid	↑	Carnitine	↓
Phenylalanine	↓	Leucine	↓	Serine	↓		
Carnitine	↓	Serine	↓	Taurine	↓		
PE(18:1/22:6)	↓	Lactic acid	↓	Leucine	↓		
Valine	↓	Niacinamide	↓	Dehydroascorbic acid	↓		
PG(16:0/16:0)	↓	Glycerophosphocholine	↓	Proline	↓		
N-Acetyl-D-glucosamine6-phosphate	↑			Lactic acid	↓		
2-Hydroxystearic acid	↑			Glutamic acid	↓		
2-Arachidonyl glycerol ether	↑			Niacinamide	↓		
Pyruvic acid	↓			Carnitine	↓		
Citric acid	↓			Glycerophosphocholine	↓		
Threonine	↓			PC(16:0/16:0)	↓		
Lactic acid	↓			Valine	↓		
Proline	↓			PG(16:0/16:0)	↓		
Leucine	↓						
Serine	↓						
Niacinamide	↓						
Glycerophosphocholine	↓						

Upward (↑) and downward (↓) arrows indicate increased and decreased metabolite levels, respectively, relative to the corresponding control group.

Table S3. DIABLO component 1 loading values of transcriptomic, lipidomic, and metabolomic features distinguishing SARS-CoV-2-infected mice from control mice.

ID	Block	Omics	Comp1_loading
Scgb1a1	transcriptomics	gene	-0.11
Cux1	transcriptomics	gene	0.88
Tmed1	transcriptomics	gene	0.21
Isg15	transcriptomics	gene	0.16
Saa3	transcriptomics	gene	0.37
Lactic acid	metabolomics	metabolite	-0.48
2-Arachidonyl glycerol ether	metabolomics	metabolite	0.45
N-Acetyl-D-glucosamine 6-phosphate	metabolomics	metabolite	0.37
Niacinamide	metabolomics	metabolite	-0.44
2-Hydroxystearic acid	metabolomics	metabolite	0.37
Vaccenic acid	metabolomics	metabolite	0.24
LysoPC(18:0)	metabolomics	metabolite	0.20
Threonine	metabolomics	metabolite	-0.02
Glycerophosphocholine	metabolomics	metabolite	-0.05
Serine	metabolomics	metabolite	-0.03
TG18:1_38:4	lipidomics	lipid	0.09
TG22:5_36:1	lipidomics	lipid	0.17
TG18:1_38:5	lipidomics	lipid	0.04
TG20:4_36:1	lipidomics	lipid	0.60
TG16:0_38:4	lipidomics	lipid	0.35
PCP-18:1_18:1	lipidomics	lipid	-0.58
PCP-16:0_18:1	lipidomics	lipid	-0.31
TG22:4_34:1	lipidomics	lipid	0.16
TG18:0_38:6	lipidomics	lipid	0.07
PCP-16:0_18:2	lipidomics	lipid	-0.12

Table S4. DIABLO loading component 1 values of transcriptomic, lipidomic, and metabolomic features distinguishing MERS-CoV-infected mice from control mice.

ID	Block	Omics	Comp1_loading
Mamdc2	transcriptomics	gene	-0.56
Rassf3	transcriptomics	gene	-0.11
Sftpc	transcriptomics	gene	-0.25
Apobec3	transcriptomics	gene	0.10
Pole	transcriptomics	gene	0.54
Ccl2	transcriptomics	gene	0.27
Shmt1	transcriptomics	gene	0.17
Areg	transcriptomics	gene	0.44
Cxcl10	transcriptomics	gene	0.12
Aplp2	transcriptomics	gene	-0.13
Niacinamide	metabolomics	metabolite	-0.71
Carnitine	metabolomics	metabolite	-0.66
Taurine	metabolomics	metabolite	-0.16
Ortophosphate	metabolomics	metabolite	-0.11
Dehydroascorbic acid	metabolomics	metabolite	-0.14
PC18:1_22:4	lipidomics	lipid	0.39
HexCer 18:1;O2_18:1	lipidomics	lipid	0.39
PC18:1_18:1	lipidomics	lipid	0.32
LPCO-18:1	lipidomics	lipid	0.30
PC22:6_22:6	lipidomics	lipid	0.29
PI20:4_20:4	lipidomics	lipid	0.24
LPCP-18:0	lipidomics	lipid	0.29
TG16:1_35:1	lipidomics	lipid	-0.19
PE18:2_22:4	lipidomics	lipid	0.12
Cer18:1;O2_18:1	lipidomics	lipid	0.25
PC16:1_18:1	lipidomics	lipid	0.20
PC18:1_18:2	lipidomics	lipid	0.15
PC18:2_22:4	lipidomics	lipid	0.22
HexCer 18:1;O2_20:1	lipidomics	lipid	0.15
PE20:1_22:4	lipidomics	lipid	0.12
TG16:0_33:1	lipidomics	lipid	-0.10
HexCer 18:1;O2_22:1	lipidomics	lipid	0.02
PC18:1_18:3	lipidomics	lipid	0.04
PE18:1_18:1	lipidomics	lipid	0.02
TG17:0_34:1	lipidomics	lipid	-0.02

Table S5. Result of Group-mean-centered sensitivity analysis of the SARS-CoV-2

SARS-CoV-2	Number of edges	Pooled median $\|\rho\|$	Residual median $\|\rho\|$	Number direction retained	%_direction retained	Number residual $\|\rho\|\geq0.5$	%_residual $\|\rho\|\geq0.5$
**Overall**	132	0.747	0.664	102	77.27	82	62.12
**Gene-Lipid**	36	0.776	0.625	26	72.22	22	61.11
**Gene-Metabolite**	33	0.723	0.673	24	72.73	20	60.61
**Metabolite-Lipid**	63	0.743	0.695	52	82.54	40	63.49

Spearman correlations were recalculated after subtracting the corresponding group mean from each feature value. The table reports the total number of selected edges, the median absolute correlation coefficient before adjustment (pooled median $|\rho|$) and after adjustment (residual median $|\rho|$), the number and percentage of edges retaining the original correlation direction, and the number and percentage of edges maintaining residual $|\rho| \geq 0.5$. Results are presented for the overall network and by inter-omics edge type. ρ, Spearman's rank correlation coefficient.

Table S6. Result of Group-mean-centered sensitivity analysis of the MERS-CoV

MERS-CoV	Number of edges	Pooled median $|\rho|$	Residual median $|\rho|$	Number direction retained	%_direction retained	Number residual $|\rho|\geq0.5$	%_residual $|\rho|\geq0.5$
**Overall**	333	0.811	0.617	226	67.87	201	60.36
**Gene-Lipid**	199	0.839	0.646	134	67.34	124	62.31
**Gene-Metabolite**	49	0.755	0.617	27	55.10	26	53.06
**Metabolite-Lipid**	85	0.783	0.573	65	76.47	51	60.00

Spearman correlations were recalculated after subtracting the corresponding group mean from each feature value. The table reports the total number of selected edges, the median absolute correlation coefficient before adjustment (pooled median $|\rho|$) and after adjustment (residual median $|\rho|$), the number and percentage of edges retaining the original correlation direction, and the number and percentage of edges maintaining residual $|\rho| \geq 0.5$. Results are presented for the overall network and by inter-omics edge type. ρ, Spearman's rank correlation coefficient.
